# Dynamic Hippocampal CA2 Responses to Contextual Spatial Novelty

**DOI:** 10.3389/fnsys.2022.923911

**Published:** 2022-08-08

**Authors:** Guncha Bhasin, Indrajith R. Nair

**Affiliations:** Division of Systems and Behaviour Neuroscience, National Brain Research Centre (NBRC), Manesar, India

**Keywords:** spatial novelty, hippocampal CA2, spatial memory and navigation, place cells, *in vivo* electrophysiology, tetrode recordings

## Abstract

Hippocampal place cells are functional units of spatial navigation and are present in all subregions: CA1, CA2, CA3, and CA4. Recent studies on CA2 have indicated its role in social and contextual memories, but its contribution to spatial novelty detection and encoding remains largely unknown. The current study aims to uncover how CA2 processes spatial novelty and to distinguish its functional role towards the same from CA1. Accordingly, a novel 3-day paradigm was designed where animals were introduced to a completely new environment on the first day, and on subsequent days, novel segments were inserted into the existing spatial environment while the other segments remained the same, allowing us to compare novel and familiar parts of the same closed-loop track on multiple days. We found that spatial novelty leads to dynamic and complex hippocampal place cell firings at both individual neuron and population levels. Place cells in both CA1 and CA2 had strong responses to novel segments, leading to higher average firing rates and increased pairwise cross correlations across all days. However, CA2 place cells that fired for novel areas had lower spatial information scores than CA1 place cells active in the same areas. At the ensemble level, CA1 only responded to spatial novelty on day 1, when the environment was completely novel, whereas CA2 responded to it on all days, each time novelty was introduced. Therefore, CA2 was more sensitive and responsive to novel spatial features even when introduced in a familiar environment, unlike CA1.

## Introduction

The discovery of place cells in the hippocampus more than a quarter of a century ago (O’Keefe and Dostrovsky, 1971) firmly established this region as the locus of the cognitive map in the brain essential for successful spatial memory and navigation. While the major hippocampal subregions (CA1, CA3, and DG) have been studied extensively and their respective roles in spatial navigation are well-characterized (Wilson and McNaughton, 1993; Zhang et al., 1998; Leutgeb et al., 2007; Mizuseki et al., 2012; Kim et al., 2020), CA2 has largely been left out of the spotlight and termed as a mere “transition zone” with no functional contribution of its own towards this map (Ishizuka et al., 1995). However, interest in CA2 has increased in the last decade, with notable findings examining the structure, function, and unique connectivity patterns of CA2, which have revealed it to be a key player in the hippocampal circuitry. This research has highlighted CA2’s important contributions to memory and social information processing (Kohara et al., 2014; Stevenson and Caldwell, 2014; Mankin et al., 2015; Dudek et al., 2016; Kay et al., 2016); however, its distinct role in spatial information processing is yet to be properly realized.

A key role of spatial exploration is successfully detecting and encoding novel stimuli (contextual or social) and integrating them with familiar stable spatial maps of an environment, which is thought to allow for continual upgrading of stored memories. CA1 has been characterized as a broadcaster of a novelty signal in the hippocampus (Larkin et al., 2014), and disruption of EC–CA1 pathway leads to impairment in spatial novelty detection (Vago and Kesner, 2008). Moreover, CA1 is uniquely poised to be neuromodulated through diverse circuitries by both CA3 and CA2 (Mankin et al., 2015) *via* the “classic” trisynaptic pathway (EC to DG to CA3 to CA1) and the recently discovered disynaptic pathway [EC(II) to CA2 to CA1] (Chevaleyre and Siegelbaum, 2010; Kohara et al., 2014), respectively. While the CA3–CA1 pathway has been extensively studied with respect to spatial navigation and memory, with some even looking at CA3–CA2 place cell topography (Lee et al., 2015; Lu et al., 2015), CA2–CA1 studies are far and few in between (Mankin et al., 2015; Alexander et al., 2016). The mutual inhibitory relationship between CA3 and CA2, controlled by feed-forward circuitry (Chevaleyre and Siegelbaum, 2010; Kohara et al., 2014) and limited plasticity (Zhao et al., 2007) invites the exciting hypothesis that there is a competition for active control of CA1 activity between these two areas.

Recent studies have shown that CA2 plays a role in social and novel contextual information processing (Hitti and Siegelbaum, 2014; Mankin et al., 2015; Alexander et al., 2016), and that it has a relatively “flexible spatial code” when compared to CA1 and CA3, since it can update its spatial representations in response to contextual changes that are social or novel in nature. Moreover, only dorsal CA2 projections to the ventral CA1 are required for social memory (Meira et al., 2018), whereas its projections to dorsal CA1 play a role in novel object recognition (Raam et al., 2017), working memory, and sequential firing patterns in CA1 (MacDonald and Tonegawa, 2021). Additionally, axons of CA2 basket cells extend to all three CA fields of the hippocampus, providing feedback inhibition to CA3 and feed-forward inhibition to CA1, making it uniquely poised to coordinate and control the hippocampal circuitry (Mercer et al., 2007). Therefore, understanding CA2–CA1 connectivity, dynamics, and interplay is imperative for elucidating memory-driven hippocampal responses.

Keeping the above in mind, the primary goal of the present study is to tease out specific contributions of CA1 and CA2 towards detection of novel spatial stimuli, especially when a novelty is introduced in an otherwise stable and relatively familiar environment. To meet this end, *in vivo* multiunit recordings were performed in the rat hippocampus to examine and distinguish between neuronal firing responses of CA1 and CA2 to spatial novelty detection. A novel 3-day paradigm where the rats experienced varying degrees of spatial familiarity and novelty with each successive day as the track was elongated and geometrically reshaped, was specifically designed for this aim. Across days (day 1 vs. day 2 vs. day 3) and within day comparisons (days 2 and 3: novel vs. familiar) between CA1 and CA2 ensemble populations were made with respect to individual neuronal responses such as average firing rates, pairwise cross correlations between familiar and novel cell pairs and spatial information scores; and ensemble responses through population correlation matrices. As an outcome, the ensemble activities of neural populations in CA1 and CA2 were estimated and interpreted to tease out the role of the CA1–CA2 system in various aspects of novelty detection and assimilation across days during a dynamically evolving spatial navigation task.

## Materials and Methods

All the procedures (animal care, surgical procedures, and euthanasia) were performed in accordance with NIH guidelines and were approved by the Institutional Animal Ethics Committee (IAEC) of National Brain Research Centre at Manesar, Haryana, constituted by the Committee for the Purpose of Control and Supervision of Experiments on Animals (CPCSEA), Government of India.

### Animal Handling and Surgical Procedures

Long–Evans rats aged 5–6 month old (*n* = 4, male) were housed individually in a reversed light-dark (12:12 h) cycle, and the experiments were carried out during the dark phase of the cycle. Prior to surgeries, all the animals were handled by the experimenter and kept on the pedestal (used for moving tetrodes post-surgery) for 30–60 min daily for a week to ten days to make the animals comfortable with its surroundings. All surgical procedures were performed under aseptic conditions. The rats were initially anesthetized using ketamine (administered at 60 mg/kg b.w.) and xylazine (administered at 8 mg/kg b.w.) and subsequently shifted to gaseous anesthesia using isofluorane for the rest of the surgery. A custom-built recording device (Microdrive) contained inside a dual bundle was made entirely from scratch in the laboratory and contained 20 independently movable tetrodes (each bundle consisting of 9 recording tetrodes +1 reference tetrode). This drive was then surgically implanted over the right hemisphere 3.5–3.7 mm posterior to the bregma and 1.7–1.8 mm lateral to the midline to simultaneously access different regions of the hippocampus (CA1 and CA2).

### Post-surgical Procedures and Animal Training

The rats were given a post-surgery recovery period of 7–10 days where post-surgical care was provided by the experimenter. To relieve pain, meloxicam was administered intramuscularly (1 mg/kg b.w.) on the day of the surgery and through the oral route (1 mg/kg b.w.) during the post-recovery period. During the post-surgical care, food and water were provided *ad libitum*. If required, the animals also received topical ointments: LOX (containing lignocaine hydrochloride gel) and Betadine around the circumference of the micro-drive implanted. Following post-surgical recovery, the tetrodes were slowly advanced, targeting CA1 and CA2 regions of the hippocampus over a period of 10–15 days by keeping the rats on a pedestal next to the recording system. During this period, the rats were also trained in the adjacent behavioral room to run clockwise, seeking chocolate sprinkles placed at random locations on a centrally placed black circular track (10 cm wide, elevated 90 cm from floor level) for 30 min/day for 8–10 days. The behavior room was covered with circular plain black curtains at its perimeter and had no other cues (proximal or distal) of any kind. During training and subsequent experimental recordings, the rats were maintained at 85% of their free-feeding weights.

### Electrophysiology and Recording

A 17-μm platinum-iridium wire from California Fine Wire (United States) was used to make the tetrodes, and the tips of individual wires of the tetrodes were electroplated with a platinum black solution (Neuralynx Inc., United States) to 100–150 kΩ with 0.2 μA current. Multi-tetrode electrophysiological recordings were carried out using a 96-channel data acquisition system (Digital Lynx 10S; Neuralynx Inc., United States) by amplifying the signals through a headstage preamplifier (Neuralynx Inc., United States). The micro-drive was fitted with an EIB-27 board at its center, which then connected to the headstage preamplifier HS-27, which was in turn connected to the commutator using HS-27 tethers. The commutator was connected with recording cables to the 96-channel Digital Lynx 10S data acquisition system (Neuralynx Inc., United States). The units were recorded against a reference electrode from that particular bundle of the dual–bundle micro-drive, which was present in a cell-free zone in the brain (the “silent zone” above the hippocampal pyramidal layer) by filtering the signal between 600 Hz and 6 kHz. Spike waveforms above the threshold of 40 μV were sampled at 32 kHz for 1 ms. Local field potentials (LFPs) were recorded against a ground screw anchored to the skull above the frontal cortex, filtered between 0.1 Hz and 1 kHz, and continuously sampled at 4 kHz. Recording signals with highest signal-to-noise ratio possible are imperative for *in vivo* studies and are highly dependent on minimizing sources of noise through appropriate reference channels. Therefore, spike units were referenced against a reference electrode inside the brain, in a cell free zone as it matched the recording arrays in terms of geometry, impedance value, and least physical separation, thereby providing the best signals (Kovacs, 1994; Ludwig et al., 2006). LFPs, on the other hand, were differentially referenced *via* a ground screw placed on top of the dural surface above the frontal cortex and distal to the actual recording site (i.e., the hippocampus), because the difference between the voltage of noise at the reference and micro tetrode sites is increased (Webster, 1998; Horsch and Dhillon, 2004). However, LFPs were not analyzed in this study. Instead, they were only used for visually observing sharp wave ripples (SWRs) that are a signature of hippocampal LFPs. The position and head direction of the animals were tracked with red and green LED lights attached to the headstages and captured through a color CCD camera that was mounted at the ceiling, central in position to the room (CV-S3200, JAI Inc., San Jose, CA, United States) at 25 Hz.

### Histological Procedures and Identification of Recording Sites

After successful completion of the electrophysiological experiments, marker lesions were performed on few selected tetrodes by passing current at 10 μA for 10 s. The rats were transcardially perfused the next day with 4% formalin solution, and the brain was extracted and stored in 30% sucrose-formalin until it sank in the solution. The brains were then sectioned in the coronal plane (40 μm thick), mounted, and stained with Nissl’s staining using 0.1% Cresyl violet. Images of serial hippocampal sections were captured with a Leica DFC265 digital camera attached to a Leica M165-C stereo microscope and saved as TIFF files. Images of sections showing clear tetrode traces in CA1 and CA2 were taken again with an Olympus microscope (BX51) for better clarity and resolution at a higher magnification (4×). The distance from the midline to the tetrode track markings were measured from the serial sections and plotted on Excel spreadsheets to visualize the configuration of all tetrode tracks. The tetrodes were then identified by comparing this configuration with the arrangement of tetrodes in the micro-drive and cross-verifying with the marker lesions as reference. Depth reconstruction of tetrode tracks was performed to identify the brain region in which the cells were recorded on each day based on the distance from the bottom tip of the tetrode by taking into account 15% shrinkage of tissues due to histological processing. Positions of all 20 tetrodes in various sub fields of the hippocampus in the dual bundle were identified this way (including the 2 reference channels). Place cell recordings from tetrodes identified in CA3 or at the CA2/CA3 and CA1/CA2 borders were not used further for any analysis (Supplementary Figure 3).

### Experimental Paradigm

**FIGURE 1:**
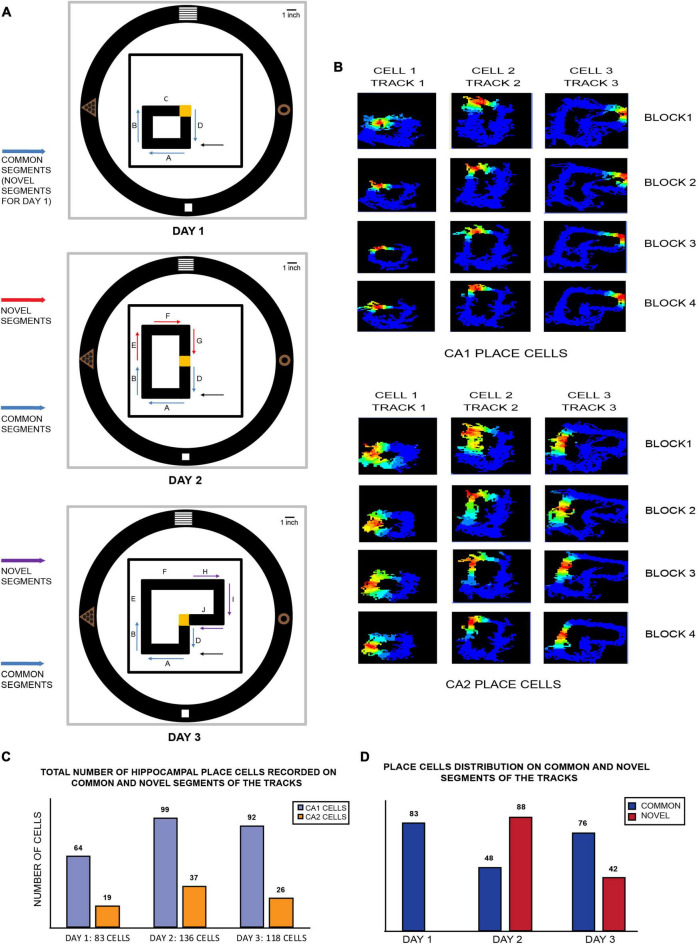
**(A)** Schematic of various closed loop tracks used in the 3-day novel experimental paradigm: on day 1, a square track of four equal arms was placed in one corner of the room (lower left quadrant of room). Arms A, B, and D remain a part of tracks on all days and are subsequently termed as “common segments” on all 3 days (or “novel segments” for day 1 since the track is novel for the animals on the first day). A reward corner (at the junction of arms C and D, indicated in yellow) is where the animals got one chocolate sprinkle after completion of every lap (clockwise). The position of this reward corner remained consistent throughout all the 3 days. On day 2, arm C is removed, and the existing track is extended to double its length. The arms introduced in the track (arms E, F, and G) are termed “novel segments” (indicated by red arrows). On the third day, arm G is removed and arms H, I, and J from day 2 are added to the existing track. The novel arms (indicated by purple arrows) are termed “novel segments,” arm C from day 1, the middle arms (arms E and F) from day 3 and the reward corner are not used for any comparative analysis to maintain consistency of size, length and geometry for the remaining tracks that are used for comparison. The remaining common and novel segments from all the tracks are similar in length and shape (C shape) and can be thus used for a myriad of comparisons against each other. The placement of reward corner (yellow corner), starting of laps on every block recording across days (arm A), along with four perpendicularly placed distal visual cues on the wall is consistent across all tracks on all days. Black arrows on all days indicate the starting point of all clockwise laps that the rats ran in all block recordings. **(B)** Color-coded firing rate maps of CA1 (top) and CA2 (bottom) place cells firing on different tracks from each day (three different place cells from CA1 and three from CA2). Each day recording consisted of 4 block sessions termed as block 1, block 2, block 3, and block 4, consisting of 20 clockwise laps each. Both CA1 and CA2 place cells maintained the same firing position during all the four blocks, as depicted in each column. **(C)** Total number of CA1 and CA2 place cells recorded on common and novel segments (combined) of each track on all days. **(D)** Distribution of hippocampal place cells according to the part of the track they fired for on each day. Cells that fired for common segments are indicated in blue while those that fired on novel segments on days 2 and 3 are in red. On day 2, majority of place cells fired for the novel segment of the track as opposed to the common segment, while a reverse distribution was observed on day 3.

At the beginning of the experiment, four distinct-shaped visual cues were hung over the black curtains at 90° to each other. A large 1 ft × 1 ft black square platform was kept in the center of the room and elevated 90 cm above the ground, over which all the differently shaped, closed loop plain black tracks (10 cm wide) were subsequently introduced each day, all elevated 15 cm above the square platform. On day 1, a small square black track (45 cm × 45 cm) was kept on the lower left quadrant of the square platform. This track was divided into four equal length arms: A, B, C, and D. The corner junction of C and D was covered with yellow sandpaper (10 cm × 10 cm), which served as the location of reward for the rat (a single chocolate sprinkle) each time it completed a clockwise lap. The position of this corner was the center of the behavior room and remained constant throughout the length of the experimental paradigm. On subsequent days, this track was elongated, and its geometry was modified such that the rats experienced varying degrees of relative spatial familiarity and novelty. Moreover, arms A, B, and D remained constant for all the three tracks and were subsequently termed “common segments” for all days. On day 2, the square track from day 1 was elongated into a rectangle-shaped black track (45 cm × 80 cm) such that the track now occupied the lower and upper left side of the square platform (two quadrants). Arm C was removed, and arms E, F, and G were added to the existing track, making arms A, B, and D the “common segments” and arms E, F, and G the “novel segments” of this track. On the third day, the rectangular track from day 2 was elongated to extend to the top right quadrant of the platform and formed an L-shaped track (occupying three quadrants). Arm G was subsequently removed, and arms H, I, J were added, making them the “novel segments” for day 3, while the common segments remained the same (Figure 1A).

#### Block Sessions

All experimental days had 4 block sessions of recording consisting of 20 clockwise laps each (except day 1 of 1 rat, which had 3 sessions) termed as blocks 1, 2, 3, and 4. This novel paradigm was designed such that at each elongation of the track across days, the rats took a longer route to complete each lap. This was conducted so that the rats could experience a completely novel environment on day 1 (0% familiarity, 100% novelty) and varying degrees of familiarity and novelty on day 2 (50% familiarity, 50% novelty) and day 3 (67% familiarity and 33% novelty) of the track environment. Except for the change in length and, thus, shape of the track, the relative position of distal visual cues, the position of reward corner, the entry point of the rats on the track (arm A, indicated by block arrow on all days in Figure 1A), and the direction of running (clockwise) laps all remained constant throughout the experiment. Each block session was interleaved with 10–15 sec of break where the rats were removed from track and put in a box to dispense their sense of direction and orientation. Then, they were released on the same starting position on the track (arm A) for the next block session. Upon completion of all the four experimental block sessions, the track was wiped clean with 70% ethanol to clear off any traces that could act as potential cues for the next day of recording.

#### “Within Day” and “Across Days” Comparisons

**FIGURE 2:**
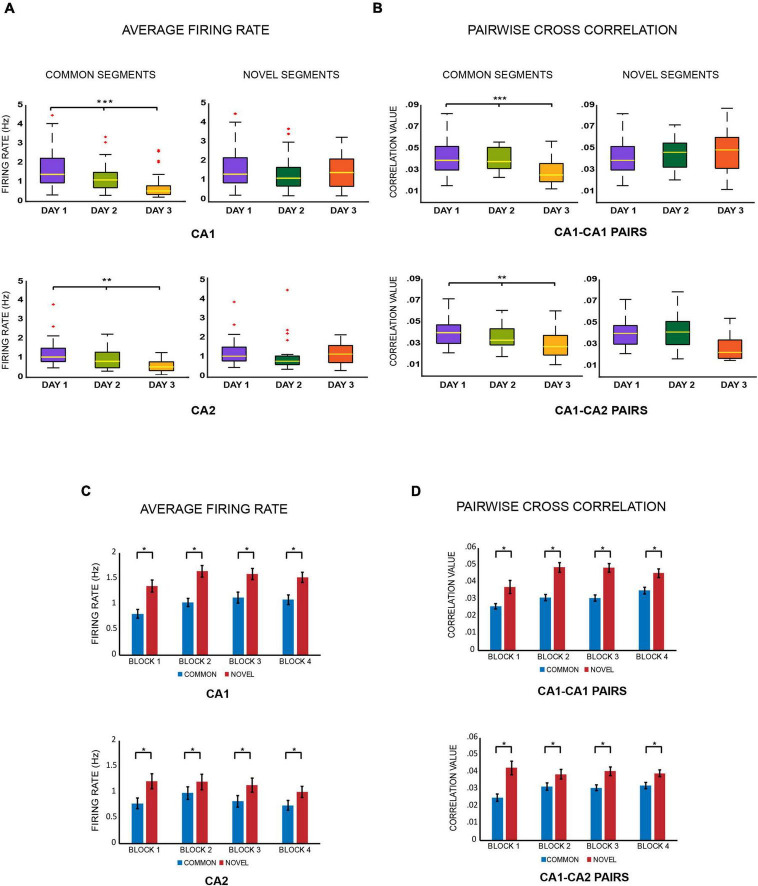
**(A)** Average firing rates. Boxplots of average firing rates of CA1 and CA2 place cells were constructed for comparing common and novel segments of the tracks across days. As the same spatial area got more familiar, the firing rates of place cells firing for that area decreased over days (*** indicates *p* < 0.005, ** indicates *p* < 0.005 and * indicates *p* < 0.05) (Kruskal–Wallis test: common segments, CA1: *p* < 0.0001; CA2: *p* = 0.0034) but not for novel areas (Kruskal–Wallis test: novel segments: CA1 cells: *p* = 0.259; CA2 cells: *p* = 0.25). **(B)** Pairwise cross correlations. Boxplots for pairwise cross correlations of CA1–CA1 and CA1–CA2 cell pairs were also compared for familiar and novel areas across days and depicted the same trend. As the same spatial area got more familiar, pairwise cross correlations of both CA1–CA1 and CA1–CA2 cell pairs firing for that area decreased over days (Kruskal–Wallis test: common segments: CA1–CA1 pairs: *p* = 0.00006; CA1–CA2 pairs: *p* = 0.0009) but not for novel areas, where the values remained similar and comparable (Kruskal–Wallis test: novel segments: CA1–CA1 pairs: *p* = 0.485, CA1–CA2 cell pairs: *p* = 0.078). **(C)** Average firing rates – For day 2 and 3, the average firing rates for place cells firing for novel segments were combined and compared against those that were firing for common segments. This was conducted for all the four recording blocks separately. For all blocks, the average firing rates for novel segments were higher than those for common segments for both CA1 and CA2 ensembles (Jonckheere-Terpstrsa test: common vs. novel segments: CA1 cells: *p* = 0.0104, CA2 cells: *p* = 0.01). **(D)** Pairwise cross correlations – The same comparisons were extended to CA1–CA1 and CA1–CA2 cell pairs, whereby the same trend was observed, where correlation for cell pairs firing for novel segments were higher than those firing for common segments of the track across all 4 blocks (Jonckheere-Terpstrsa test: CA1 vs. CA2 cells: common segments: *p* = 0.0104, novel segments: *p* = 0.02).

**FIGURE 3:**
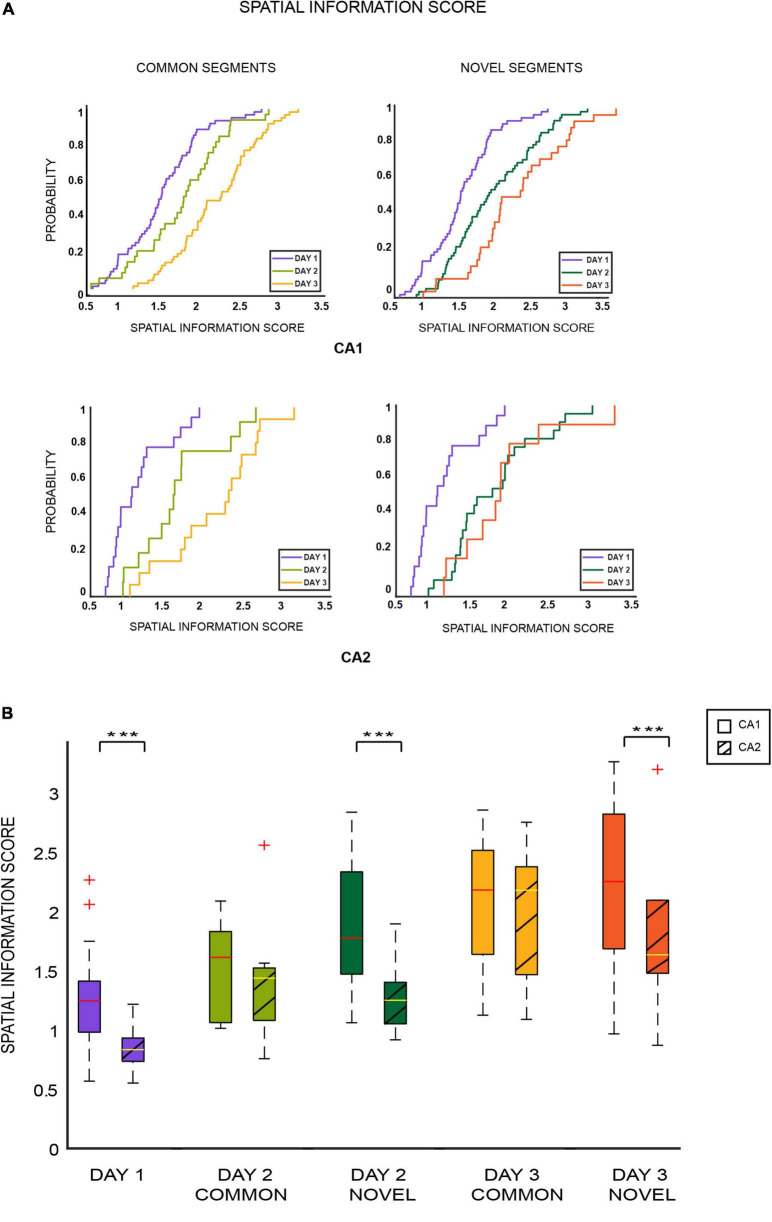
**(A)** Cumulative distribution plots (EDCFs) for CA1 and CA2 place cells across days for common (left column) and novel segments (right column). For both sets of comparisons, spatial information scores for both ensembles increased over days, as overall familiarity of the environment increased (*** indicates *p* < 0.0005) (two-sample Kolmogorov–Smirnov test: common segments: CA1 cells: *p* < 0.01, CA2 cells: *p* < 0.02; novel segments: CA1 cells: *p* < 0.001, CA2 cells: *p* < 0.005 except for RUN2new/RUN3new *p* = 0.77). **(B)** Spatial information scores were calculated and plotted as boxplots for CA1 and CA2 cells separately and compared across days for different segments of the tracks with respect to familiarity and novelty. It was observed that spatial information scores were lower for CA2 place cells that coded for novel areas of the track (day 1, day 2 novel, and day 3 novel) in comparison to CA1 place cells coding for the same novel space. However, information scores for both ensembles remained similar and comparable for familiar areas (day 2 common and day 3 common) [Mann–Whitney test: novel segments (days 1, 2, and 3): CA1 vs. CA2: *p* = 0.00004; common segments (days 2 and 3): CA1 vs. CA2: *p* = 0.2124]. This indicates poor spatial coding in CA2 place cells for novelty but not familiarity in comparison to CA1.

Data from arm C from day 1 and arms E and F from day 3, as well as any place cell that fired for the reward area on all days were not used in any analysis to maintain length and geometric accuracy of the remaining tracks for subsequent comparisons. The remaining arms from all the tracks that constitute the common segments (arms A, B, and D) and the novel segments on day 2 (arms E, F, and G) and day 3 (arms H, I, and J) were all C-shaped tracks that were similar in shape and size and could thus be used for comparative analysis with each other. Therefore, not only could the common segments be compared with each other across days, the common segment from day 1 could also be compared against novel segments from subsequent days as well, since the common segment on day 1 was 100% novel itself. Moreover, common segments from days 2 and 3 could be also compared with their corresponding novel segments within that day. This myriad of combinative comparisons allowed for comparing and analyzing spatial familiarity and novelty on multiple time scales, resulting in the following:

1. Within day comparisons (block comparisons): comparative analysis across all the four blocks within each day, as block 1 is most novel and block 4 is most familiar session for the day. This could also be carried out separately for common and novel segments of a particular track (on days 2 and 3) to observe how both segments become increasing familiar throughout the day (Figure 5B and Supplementary Figures 4A, 5A, 6A).
2. Within day comparisons (familiar vs. novel): for days 2 and 3, a comparison between the common and novel segments over the four blocks (or averaged together) could be made (Figures 2C,D and Supplementary Figures 4B, 5B, 6B).

3. Across days comparisons: the common segments could be compared across days 1, 2, and 3, as they became increasingly familiar over the days. The common segment from day 1 could also be compared against the novel segments from days 2 and 3, since all the three segments were 100% novel on the day they were introduced in the environment (Figures 2A,B, 3A,B, 5B,C).

## Data Analysis

All the quantitative analyses of data were performed with custom-written software on MATLAB (R2013a, 2018, 2020a),^1^ as described below. The significance value (alpha) of all statistical tests performed was set at 0.05 unless specified otherwise. All violin plots were made with the GraphPad Prism 9.0 software.^2^

### Isolation and Classification of Single Units

Isolation of single units was performed manually with custom-written spike-sorting software WinClust (Siegel et al., 2008; Savelli et al., 2017). Cells were isolated based on spike parameters such as peak amplitude and energy of waveforms recorded on all four wires of the tetrode. Offline spike sorting of multiple clusters recorded from the same tetrode was conducted by principal component analysis (PCA) and isolating each cluster in various projections (six projections for four wires of each tetrode: 1/2, 1/3, 1/4, 2/3, 2/4, and 3/4). Based on their isolation quality (distance from the background and separation from other clusters), waveform shape, interspike interval (ISI), and potential overlap between neighboring clusters present, the units were qualitatively rated on a scale ranging from 1 to 5 (1, very good; 2, good; 3, fair; 4, marginal; 5, poor), and units rated “fair” and above were used further for data analysis (Siegel et al., 2008; Savelli et al., 2017; Nair et al., 2022). Cluster ratings were independent of spatial properties of the unit isolated. Clusters that had more than 1% spike less than ISI inter spike interval of 3 ms were discarded. Putative interneurons (units with mean firing rate >10 Hz) were excluded from analysis. Only units identified as place cells were used in this study

### Linearization of Tracks

Position data from all the four block sessions of a particular day for each rat were loaded in MATLAB, and outer and inner boundaries were defined, in alignment with the track by removing outliers. The position where the rats were left on the track at the beginning of each block was defined as the starting point of arm A, and subsequent arms of each track were defined clockwise. All the tracks were linearized and converted into a one-dimension track and divided into 2-cm spatial bins. Finally, a speed filter of 2 cm/s was applied to exclude stationary periods when the animals did not move.

### Defining Place Field Characteristics

#### Spatial Information Score

Spatial information score was calculated for all previously chosen clusters defined as place cells. The value of spatial information indicates the amount of information about the rat’s position conveyed by firing of a single spike from a cell (William and McNaughton, 1992) and was calculated as:


$$I=\sum_{x}\lambda \,\left(x\right)\,{\text{log}}_{2}\,\left(\frac{\lambda \,\left(x\right)}{\lambda }\right)\,p\,\left(x\right)$$

where *x* is spatial bin, λ(*x*) is the firing rate of the cell at location, λ is the mean firing rate, and *p*(*x*) is the probability of occupancy at bin *x*. A cell was classified as a place cell if its spatial information score was found to be significant (*p* < 0.05) based on cell shuffling procedure (defined below) performed on each cell individually in any of the experiment blocks recorded in a particular day.

Data shuffling procedure: an individual cell’s entire spike sequence recorded in a particular block was time-shifted with a random interval between the 20th second and 20 s before termination of that block. Spikes exceeding the total time of the block were wrapped around to be assigned to the beginning of that block to generate a new spike time sequence for that cell. This procedure was repeated 100 times for each cell separately. The 99th percentile value of the shuffled distribution of each score was taken as the threshold value for firing of that particular cell (Langston et al., 2010).

#### Average Firing Rates

Chosen clusters that fit the criteria for being defined as a place cell based on its spatial information score were subsequently loaded in MATLAB, and only clusters that fired more than 50 spikes in each block recording were eventually chosen for analysis. Firing rate was calculated as the ratio between the number of spikes and time spent in each bin. Linearized rate maps of 2-cm spatial bins were smoothed with a Gaussian smoothing function of 4 cm standard deviation and characterized as having a firing rate greater than 1 Hz over a minimum of five continuous spatial bins and an occupancy rate greater than 0.1 Hz. Place fields were further defined as having a mean firing rate between 0.1 and 5 Hz and a peak firing rate of minimum 2 Hz. A cell’s peak rate was defined as the firing rate in the bin with the highest rate on the linear track. Place field borders were defined as where the firing rate fell to less than 10% of the peak firing rate of the cell or less than 1 Hz, whichever was higher. Firing rates for place cells firing in multiple blocks were averaged for the day, across all block recordings for that particular day. Place field centers were calculated for all the place cells based on the spatial bin that had the highest firing rate for that particular cell (Dragoi and Tonegawa, 2013; Pfeiffer and Foster, 2013). Using place field centers, cells were arranged on each track for a particular day from the beginning of arm A, in the direction the rats traversed the tracks (clockwise), to the last arm of each respective track. Finally, color-coded firing rate maps of CA1 and CA2 place cells were constructed for all relevant parts of the tracks for all the 3 days (Figure 1B).

#### Pairwise Cross Correlations

Pairwise cross correlations were chosen as a characteristic to be compared in this study, since previous studies have reported that CA1 cell pairs with overlapping place fields show higher correlation and coordinated activity during high frequency events (HFEs) in novel environments and decrease with increase in familiarity (Cheng and Frank, 2008; Theodoni et al., 2018). To check if the same could be observed in this study, pairs were chosen based on their firing field location for common segments (both place cells firing on arm A, B, or D) and novel segments (both place cells firing on arms E, F, and G for day 2 and H, I, and J for day 3) separately for all the 3 days. Only cell pairs that had overlapping place fields (peak distance <15 cm) were chosen and recorded on different tetrodes (Wilson and McNaughton, 1994; Skaggs and McNaughton, 1996). The cell pairs included CA1–CA1 pairs and CA1–CA2 pairs, since CA2–CA2 pairs were too small in number for separate comparison. Correlations were computed using the xcorr function in MATLAB with a time lag of 400 ms.

#### Population Correlation Matrices

Two-dimensional population correlation matrices were constructed for CA1 and CA2 ensembles separately for each of the experimental days: days 1, 2, and 3. The mean firing rate of all the place cells were calculated for each bin of the respective tracks on which they fired on (72 bins for day 1, 106 bins for day 2, and 142 bins for day 3), resulting in an N × T matrix where N = total number of place cells and T = total number of position bins. Pearson’s product-moment correlation analysis was performed between block 1 and the remaining blocks of that day, i.e., block 1 vs. block 2, block 1 vs. block 3, and block 1 vs. block 4, creating three matrices for a particular day and further resulting in 72 × 72 bin correlation coefficient matrix for day 1, 106 × 106 bin matrix for day 2, and 142 × 142 bin matrix for day 3. All the correlation matrices comprise of Pearson correlation values acquired from bin-wise correlation of 1-D firing rate arrays. The same was also conducted with respect to block 4 (block 1 vs. 4, block 2 vs. 4, and block 3 vs. 4). A band of high correlation (in yellow) indicates coherent firing and spatial stability from one block to another (successive or non-successive blocks), while black refers to lowest correlation (Neunuebel et al., 2013; Nair et al., 2022). The mean correlation was calculated and quantified at the diagonal of each matrix and then compared using full width at half maximum (FWHM) instead of 1D polar plots because of the non-circular nature of the tracks (Deshmukh, 2021). For each day, population matrix comparisons were carried out against block 1 (most novel recording session of each day) and against block 4 (most familiar session of each day) for both ensembles. This resulted in five combinatory matrices for each day: block 1 vs. block 2, block 1 vs. block 3, block 1 vs. block 4, block 2 vs. block 4, and block 3 vs. block 4 for CA1 and CA2 populations separately. The same matrices were also created on subsequent days, resulting in 15 (5 × 3) matrices for CA1 and 15 more for CA2.

## Results

A total of 427 place cells (from *n* = 4 animals) from CA1 and CA2 were recorded over 3 days of the experimental paradigm. Excluding cells that fired for the reward area each day, a total of 288 place cells from CA1 and 94 from CA2 remained that fired for all 3 tracks. Excluding cells that fired for arms C, E, and F, a total of 83 place cells fired on day 1 (CA1: 64 cells, CA2: 19 cells), 136 cells on day 2 (CA1: 99 cells, CA2: 37 cells), and 118 cells on day 3 (CA1: 92 cells, CA2: 26 cells) for all common and novel segments (Figure 1C). Furthermore, the above mentioned place cell distributions of CA1 and CA2 populations were combined and collated according to novel and common areas on the track on days 2 and 3. On day 2, it was observed that most place cells fired for the novel segment of the track compared to the common segment (novel segment: 88 cells, common segment: 48 cells). On day 3, however, the reverse was observed as a higher number of place cells fired for the common segment (common segment: 76 cells, novel segment: 42 cells) (Figure 1D and Supplementary Figure 1B).

### Average Firing Rates and Pairwise Cross Correlations Decrease Over Time for Spatially Familiar but Not Novel Areas

Average firing rates and pairwise cross correlations were computed for hippocampal place cells separately over all the segments of the tracks for all the days. Average firing rates were calculated for CA1 and CA2 place cells separately, as described in detail in section “Materials and Methods.” Pairwise cross correlations were computed for common segments on day 1 and for familiar cell pairs and novel cell pairs, i.e., both cells coding for common segments or both coding for novel segments of the respective tracks for days 2 and 3.

Average firing rates for place cells that fired over common segments were compared across days for CA1 and CA2 populations separately. Both populations showed a decrease in average firing rates from days 1 to 3 (Kruskal–Wallis test: common segments, day 1 vs. day 2 vs. day 3: CA1 cells: *p* < 0.0001; CA2 cells: *p* = 0.0034). However, the same was not observed for novel segment comparisons (Kruskal–Wallis test: novel segments, day 1 vs. day 2 vs. day 3: CA1 cells: *p* = 0.259; CA2 cells: *p* = 0.25) (Figure 2A). The above observations indicate that the average firing rates of hippocampal CA1 and CA2 place cells decreased across days with increasing familiarity of the spatial environment, but remained similar and comparable for novel spaces. Moreover, this trend was also observed across all the four blocks for common segments (refer to Supplementary Figure 4A).

The same comparisons were carried out for pairwise cross correlations between CA1–CA1 cell pairs, and CA1–CA2 cell pairs separately, for common and novel segments across days, resulting in the same trend seen for average firing rates. While correlations decreased over days for cell pairs firing on common segments (Kruskal–Wallis test: common segments, day 1 vs. day 2 vs. day 3 : CA1–CA1 cell pairs: *p* = 0.00006; CA1–CA2 cell pairs: *p* = 0.0009) (Figure 2B), no discernable differences were observed for cell pairs that fired on novel segments (Kruskal–Wallis test: novel segments, day 1 vs. day 2 vs. day 3: CA1–CA1 pairs: *p* = 0.485; CA1–CA2 cell pairs: *p* = 0.078) (Figure 2B). This trend was also observed across all the four blocks for common segments (refer to Supplementary Figure 5A).

Next, for days 2 and 3, average firing rates from place cells firing for common segments from both days were combined and compared with those firing for novel segments from both days, for CA1 and CA2 separately. Furthermore, they were compared for each block individually to tease apart differences, if any should emerge, as familiarity also increases within the same day, traversing from block 1 (first 20 laps) to block 4 (60th–80th lap). Although no differences emerged in firing rate changes between blocks, average firing rates for novel areas remained consistently higher than familiar areas across all four blocks for both ensembles (Figure 2C and refer to Supplementary Figure 4B) (Jonckheere-Terpstrsa test: common vs. novel: CA1 cells: *p* = 0.0104; CA2 cells: *p* = 0.01). The same comparison was extended to comparing pairwise cross correlations between CA1–CA1, and CA1–CA2 cell pairs, and the same trend was observed (Figure 2D and refer to Supplementary Figure 5B) (Jonckheere-Terpstrsa test: common vs. novel: CA1–CA1 pairs: *p* = 0.0104; CA1–CA2 pairs: *p* = 0.02).

The above comparisons indicate that the CA1 and CA2 populations respond similarly in modulating their average firing rates and pairwise cross correlations when coding for familiar and novel space. However, despite their similar responses, overall average firing rates for CA2 ensembles were lower those for CA1, irrespective of the track segment they coded for on days 2 and 3 (Jonckheere-Terpstrsa test: common segments: CA1 vs. CA2 cells: *p* = 0.041, novel segments: CA1 vs. CA2 cells: *p* = 0.0104). However, the cross correlations between CA1–CA1 and CA1–CA2 cell pairs remained comparable for both familiar and novel areas (Jonckheere-Terpstrsa test: common segments: CA1–CA1 vs. CA1–CA2 pairs: *p* = 0.386, novel segments: CA1–CA1 vs. CA1–CA2 pairs: *p* = 0.124). We speculate that had there been enough CA2–CA2 cell pairs, a difference would have probably been observed, as was done for firing rates between the two cell populations.

### CA2 Place Cells Have Lower Spatial Information Content Than CA1 Place Cells Coding for Novel Space

Spatial information scores of CA1 and CA2 place cells were calculated and compared for various segments of the tracks on all the days. Comparisons similar to those conducted for firing rates and cross correlations were carried out for spatial information scores of common and novel segments for both ensembles. It was observed that spatial information score increased with increase in overall familiarity of the environment for both common (day 1 vs. day 2 vs. day 3) and novel (day 1 vs. day 2 vs. day 3) segment comparisons (ECDF plots: Figure 3A) (two-sample Kolmogorov–Smirnov test: common segments: CA1 cells: *p* < 0.01, CA2 cells: *p* < 0.02; novel segments: CA1: *p* < 0.001, CA2: *p* < 0.005 except for day 2 novel/day 3 novel, *p* = 0.77). This increasing trend was also observed across all the four blocks for the common and novel segments (refer to Supplementary Figure 6A).

This trend of increasing spatial information scores across familiar areas was the opposite of that observed for average firing rates and pairwise cross correlations. Moreover, the information scores increased for novel segments as well, as opposed to no trend (increasing or decreasing) observed for firing rates or cross correlations. This could indicate that spatial information scores respond differently to spatial novelty and familiarity compared to the hippocampal neuronal characteristics previously compared. Moreover, the same trend observed for both common and novel segments could indicate that spatial information scores are less sensitive to (or affected by) relative familiarity and novelty (as opposed to firing rates and cross correlations) but are influenced more by the overall increasing familiarity of the environment (hence the increasing values for both segments).

Next, spatial information scores were compared across common and novel segments for CA1 and CA2 populations. For this comparison, only place cells that fired in both the first (most novel block of the day) and last blocks (most familiar block of the day) were included, and their scores in these blocks were averaged across. This was conducted so differences (if any) between CA1 and CA2 coding with respect to spatial novelty could be highlighted with maximum effect. Consequently, the spatial information scores of CA1 and CA2 were similar for familiar parts of the tracks (Mann–Whitney *U* test: common segments (days 2 and 3): CA1 vs. CA2: *p* = 0.2124). However, CA2 had a lower spatial information scores for novel parts of the tracks than CA1 (Mann–Whitney *U* test: novel segments (days 1, 2, and 3): CA1 vs. CA2: *p* = 0.000042) (Figure 3B).

Thus, spatial information in CA2 place cells coding for a novel spatial area was lower in comparison to CA1 place cells coding for the same novel space. However, for familiar areas, the information scores remained similar and comparable, indicating CA2’s sensitivity to spatial novelty and its ability to distinguish relative familiar and novel spatial areas locally (within the same closed loop tracks).

### CA2 Population Has Less Consistent Spatial Firing Than CA1 Ensembles Over Time

**FIGURE 4:**
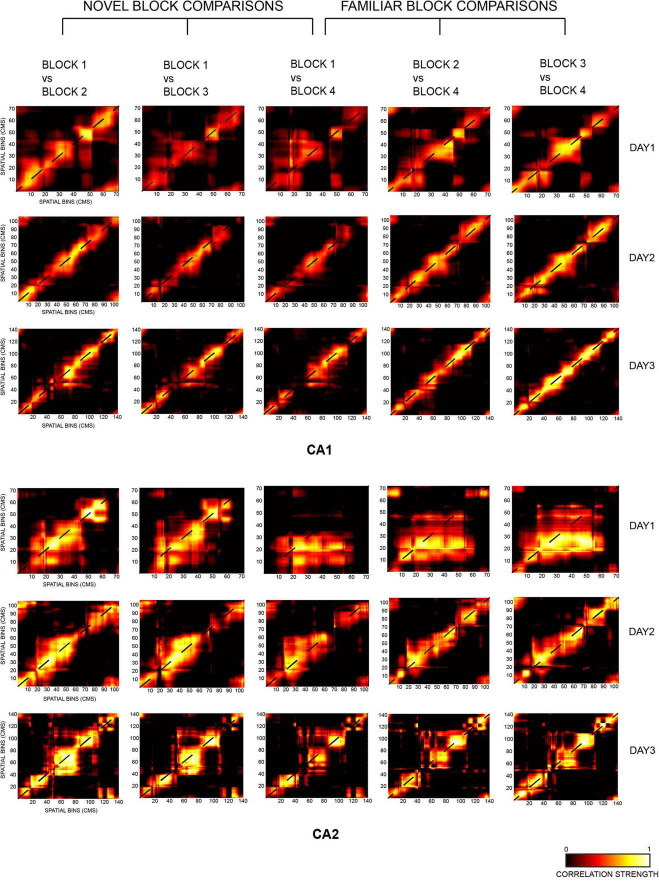
Two-dimensional population correlation matrices for CA1 and CA2 ensembles were constructed for days 1, 2, and 3 with respect to the most novel session of each day, i.e., block 1 (termed as novel block comparison, consisting of block 1 vs. block 2, block 1 vs. block 3, and block 1 vs. block 4 comparisons) and the most familiar block of each day, i.e., block 4 (termed as familiar block comparison, consisting of block 1 vs. block 4, block 2 vs. block 4, and block 3 vs. block 4 comparisons). Highest spatial correlation was seen on the diagonal of all matrices, indicated in yellow (except for block 1 vs. 4 for CA2 on day 1 due to small number of neurons recorded in block 4).

Broadening the comparisons between CA1 and CA2 to the ensemble/population level helps understand every aspect of their response to spatial coding. Thus, to measure their population responses to varying degrees of spatial novelty and familiarity each day, population correlation matrixes for each day’s recording were constructed with respect to the (a) most novel: block 1 and (b) most familiar: block 4. Spatial correlation matrices were created from population firing rate vectors in each location for each of the tracks. The firing rate vectors of block 1 were then correlated with the firing rate vectors of blocks 2, 3, and 4 for each day for CA1 and CA2 ensembles separately. These are termed as “novel block comparison.” Similarly, the firing rate vectors of block 4 were correlated with those of preceding blocks: 1, 2, and 3, termed as “familiar block comparison.” Each matrix depicts the magnitude of correlation between population vectors for each position bin on the track as a function of relative displacement between the two sessions being compared. Bands of highest spatial correlation were found on the diagonal of all matrices, termed as absolute diagonal (marked by a black line), as indicated in yellow (Figure 4). The correlation values of the 2D matrix were averaged across the diagonals of the entire matrix and converted into 1D correlation curves. The correlations were found to decrease on any other diagonal of the matrix in comparison to the absolute diagonal (Neunuebel et al., 2013). The mean correlations along the matrix diagonal were subsequently plotted for novel and familiar block comparisons for CA1 and CA2 populations separately for all days and were termed as 1D diagonal correlation plots. The peak refers to the highest mean correlation at the absolute diagonal of each 2D matrix.

**FIGURE 5:**
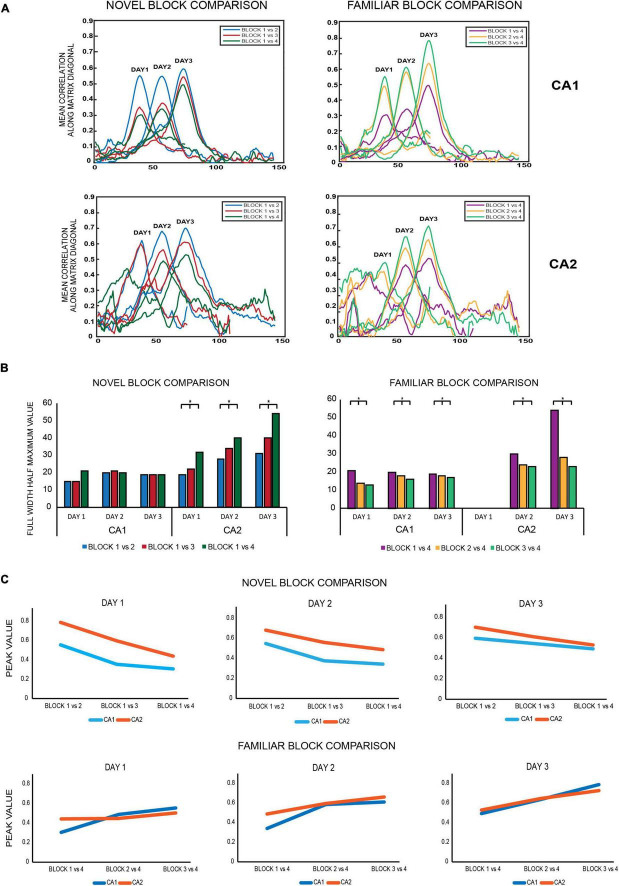
**(A)** Novel block comparison: All 2D correlation plots were converted to 1D correlation curves by averaging the correlation values of the 2D matrix along the diagonal and subsequently were termed as 1D diagonal correlation plots. All diagonal correlation plots were smoother for CA1 than for CA2. Within each day, correlation values at the diagonal were highest for the block 1 vs. 2 matrix (blue) followed by block 1 vs. 3 (red) and least for block 1 vs. 4 (dark green) for both ensembles, indicating that population correlations were highest for successive block comparisons and decreased for non-successive block comparisons as time between the blocks being compared increased. Familiar block comparisons: the same comparisons revealed the same trend as observed for novel block comparisons, with the highest correlation observed for successive block comparisons (block 3 vs. 4, indicated in light green), followed by non-successive block comparisons of block 2 vs. 4 (yellow) and least for block 1 vs. 4 (magenta) for both CA1 and CA2 ensembles. All diagonal correlation plots were again smoother for CA1 than for CA2. **(B)** Novel block comparison – The strength of spatial correlations was measured by FWHM (full-width half-maximum) values, since polar plots could not be constructed for non-circular tracks. Within each day, FWHM values for all novel block comparisons increased from successive to non-successive block comparisons for CA2 but remained similar and comparable for CA1 (Jonckheere-Terpstrsa test: CA1: *p* = 0.158, CA2: *p* = 0.022). Moreover, for each of the individual novel block comparisons, their respective FWHM values increased across days for CA2 but not for CA1 (Jonckheere-Terpstrsa test: day 1 vs. day 2 vs. day 3: FWHM novel block comparison: CA2: *p* = 0.0227, CA1: *p* = 0.412). Finally, the FWHM values were compared for each novel block comparison between CA1 and CA2 for all days, and the values were only comparable on day 1 but not on subsequent days, with CA2 having higher values (Jonckheere-Terpstrsa test: CA1 vs. CA2 FWHM values: day 1: *p* = 0.0633, day 2: *p* = 0.024, and day 3: *p* = 0.0247). Familiar block comparison – For this comparison, both CA1 and CA2 followed the same trend in the strength of their spatial correlations: highest for successive block comparisons and steady decrease for non-successive comparisons (except for day 1 for CA2 due to less number of neurons firing in the last block for day 1) (Jonckheere-Terpstrsa test: FWHM familiar block comparison: CA1 ensemble: *p* = 0.0053, CA2 ensemble: *p* = 0.0085). Moreover, for both CA1 and CA2 ensembles, each familiar block comparison values did not vary across days and instead remained comparable (Jonckheere-Terpstrsa test: all familiar block comparisons: day 1 vs. day 2 vs. day 3: CA1: *p* = 0.252, CA2: *p* = 0.275). FWHM value comparisons between CA1 and CA2 again revealed significant differences between the two for both days 2 and 3 (comparison could not be made for day 1 because of low number of CA2 cells in block 4 for that day) (Jonckheere-Terpstrsa test: CA1 vs. CA2 FWHM values: day 2: *p* = 0.0247 and day 3: *p* = 0.02). **(C)** Peak correlation values were also compared for novel and familiar block comparisons for CA1 and CA2 populations. For novel block comparisons (top), within-day responses remained the same for both ensembles on all days, with highest peak observed for successive block comparisons (block 1 vs. 2) and least for non-successive blocks (block 1 vs. 4). However, peak correlations were significantly higher for CA2 than for CA1 in all comparisons and on all days (Jonckheere-Terpstrsa test: CA1 vs. CA2 peak correlation value: day 1 vs. day 2 vs. day 3: *p* = 0.0053), although as overall familiarity increased across days, the difference between peak correlation values for the two ensembles decreased. For familiar block comparisons, on the other hand (bottom), peak values were similar and comparable for CA1 and CA2 ensembles for both within day and across days (Jonckheere-Terpstrsa test: CA1 vs. CA2 peak correlation value for day 1 vs. day 2 vs. day 3: *p* = 0.369) (* indicates *p* < 0.05). Thus, while there were differences in CA1 and CA2 population responses to novel block comparisons, both ensembles did not vary in their responses to familiar block comparisons in any way, highlighting CA2’s different responses to spatial novelty.

#### Novel Block Comparison

The diagonal correlation plots revealed the highest spatial correlation for block 1 vs. block 2 (indicated in blue), followed by block 1 vs. block 3 (red), and the lowest for block 1 vs. block 4 (green) for all the days for both CA1 and CA2 population ensembles. Thus, within a day, spatial correlation was found to be highest for successive block comparisons than non-successive block comparisons for both ensembles. The plots for CA1 were also smoother when compared to those for CA2 (Figure 5A, novel block comparison), indicating a stronger spatial correlation between CA1 unit activity than CA2 unit activity.

The strength of spatial correlation in the diagonal of novel block matrices was compared using FWHM (Deshmukh, 2021) for CA1 and CA2 over all 3 days, since polar plots could not be used for determining the strength of correlation for non-circular tracks. FWHM values for blocks 1 vs. 2, 1 vs. 3, and 1 vs. 4 were compared between CA2 and CA1 for each day, and CA2 values were found to be consistently higher for days 2 and 3 but not for day 1 (Jonckheere-Terpstrsa test: CA1 vs. CA2: day 1: *p* = 0.0633, day 2: *p* = 0.024, day 3: *p* = 0.0247). Moreover, within each day, the FWHM values for CA2 were lowest for successive block comparisons and increased as time between the blocks that were compared increased (Jonckheere-Terpstrsa test: FWHM comparison for block 1 vs. 2 V/S block 1 vs. 3 V/S block 1 vs. 4 for all days: CA2 ensemble: *p* = 0.022). However, they did not vary beyond day 1 for CA1 (CA1 ensemble: *p* = 0.158), thereby indicating that CA1 showed no spatial correlation differences for successive or non-successive novel block comparisons (Figure 5B, novel block comparison). Therefore, within a day, spatial correlations between successive blocks were consistently higher for CA2 and decreased with increase in time between non-successive blocks (as indicated by lowest FWHM values for block 1 vs. block 2). Moreover, the FWHM values for block 1 vs. 2 increased across days for CA2 but remained similar and comparable for CA1. The same was also observed for block 1 vs. 3 and block 1 vs. 4 FWHM values (Jonckheere-Terpstrsa test: day 1 vs. day 2 vs. day 3: CA2: *p* = 0.0227, CA1: *p* = 0.412) (Figure 5B, novel block comparison). Consequently, spatial correlations for CA2 decreased as more and more time passed and with increasing familiarity of the overall environment across days.

Finally, peak values from the diagonal of all the matrices were compared for CA1 and CA2 and depicted the same pattern for all the 3 days (block 1 vs. block 2 > block 1 vs. block 3 > block 1 vs. block 4). However, CA2 had a higher peak correlation value than CA1 for all the block comparisons (Jonckheere-Terpstrsa test: CA1 vs. CA2 peak correlation value: *p* = 0.0053) across days, although as overall familiarity increased, the difference between correlation values for the two ensembles decreased over days (Figure 5C, novel block comparison).

#### Familiar Block Comparison

The same comparisons were extended to familiar block comparisons, and spatial correlations were again found to be highest for successive block sessions and decreased for non-successive block comparisons: block 3 vs. block 4 (indicated in light green) > block 2 vs. block 4 (yellow) > block 1 vs. block 4 (magenta) for both ensembles (Figure 5A, familiar block comparison). Additionally, the diagonal correlation plots for CA1 were again smoother than those for CA2.

Consequently, FWHM comparisons also revealed the strongest correlations for block 3 vs. 4, followed by block 2 vs. 4 and least for block 1 vs. 4 across all days. However, this trend was observed for both CA1 and CA2 this time (except for day 1 for CA2 due to less number of neurons firing in the last block for day 1), in contrast to the novel block comparisons where only CA2 showed this trend (Jonckheere-Terpstrsa test: familiar block comparison for all days: CA1 ensemble: *p* = 0.0053, CA2 ensemble: *p* = 0.0085) (Figure 5B, familiar block comparison). Thus, for familiar block comparisons, both CA1 and CA2 followed the same trend for the strength of their spatial correlations within each day: highest for successive block comparisons and steady decrease for non-successive comparisons. Moreover, for both ensembles, FWHM values for any of the familiar block comparisons (block 3 vs. 4, block 2 vs. 4, and block 1 vs. 4) did not vary across days and instead remained comparable (Jonckheere-Terpstrsa test: day 1 vs. day 2 vs. day 3: CA1: *p* = 0.252, CA2: *p* = 0.275) (Figure 5B, familiar block comparison). However, within days 2 and 3, respective FWHM values for block 1 vs. 4, block 2 vs. 4, and block 1 vs. 4 were again higher for CA2 than for CA1, as was the trend observed for novel block comparisons (Jonckheere-Terpstrsa test: CA1 vs. CA2 familiar block comparisons: day 2: *p* = 0.024, day 3: *p* = 0.024).

Finally, a comparison of peak correlation values did not reveal a decrease from successive to non-successive block comparisons; instead, CA1 and CA2 peak correlation values remained similar and comparable across all block comparisons and for all days (Jonckheere-Terpstrsa test: CA1 vs. CA2 peak correlation value: *p* = 0.369) (Figure 5C, familiar block comparison).

The above population matrix comparisons for different combinatorial block correlations reveal interesting differences in how CA1 and CA2 process not just spatial novelty and familiarity but also the temporal aspect of an experience. In comparison to CA1, CA2 ensembles had broader diagonal correlation plots (indicated by higher FWHM values) for both familiar and novel block comparisons; however, it had higher peak correlation values only for novel block comparisons, indicating that its response to spatial novelty differs from that of CA1. This is further observed with increasing FWHM values for each of the individual novel block comparisons for CA2 but not for CA1, revealing its more robust response to spatial novelty over both time and space, with narrowest diagonal correlation plots for day 1 that got broader over days and time with overall increasing familiarity with the environment. This indicates that CA2 could be more sensitive to environmental changes, especially local changes, with respect to spatial novelty than CA1.

Furthermore, for within day comparisons, spatial correlation in CA2 ensembles is highest between successive block comparisons and decreases as a function of increasing time between the blocks being compared, for both sets of comparisons. On the other hand, CA1 exhibits this trend only for familiar block comparisons. Moreover, for novel block comparisons, CA2 FWHM values continually increased over time both within each day and for each particular block comparison across days. Conversely, this trend was only visible within each day but not across days for familiar block comparisons, indicating CA2’s role in temporal processing of spatial novelty but not spatial familiarity.

In conclusion, the above observations indicate that CA1 and CA2 process spatial familiarity similarly but not spatial novelty, and that CA2 could be more sensitive to environmental changes, especially local changes, with respect to novelty than CA1.

## Discussion

The aim of this study was to elucidate how hippocampal ensembles, particularly CA1 and CA2, dynamically evolve, modify, and code for spatial familiarity and novelty in the same environment. The specific paradigm designed for this study allowed for animals to experience varying degrees of spatial novelty and familiarity not just across days and within the same day but also within the same lap in the same closed loop tracks they traversed.

As novelty in the overall environment decreased across days, so did CA1 and CA2 average firing rates and pairwise cross correlations, whereas their spatial information score increased. For the common segments of the track, this pattern was true across days over both space and time. Ostensibly, CA2 follows a familiar pattern observed in CA1; however, more in-depth analyses revealed clear differences in their nature of spatial responses to novelty. CA2 place cells had less consistent spatial firing with each block comparison than CA1 place cells, and this effect became even greater over successive recording days. Additionally, CA2 had lower average firing rates and pairwise cross correlations for both familiar and novel areas than CA1. However, spatial information scores for CA2 were lower only for novel areas and remained similar and comparable to CA1 for familiar areas. Overall, these observations seem to indicate that both hippocampal subregions may process spatial novelty differently, and that CA2 place cells coding for a novel area do not carry as much spatial information about the environment as CA1 place cells coding for the same novel area do.

The abovementioned hippocampal neuronal responses indicate a linear relationship between average firing rates and pairwise cross correlations and an inverse relationship with spatial information scores for both hippocampal populations for spatial familiarity. This linear correlation-firing rate relationship has been extensively studied *in vitro* (de la Rocha et al., 2007), in various regions of the visual cortex (Bair et al., 2001; Cohen and Maunsell, 2009; Lin et al., 2015) and *via* computational models (Barreiro and Ly, 2017, 2018). Even more, studies have indicated that pairwise correlations that are stimulus-driven increase with firing rates, and that this positive relationship supports information processing and stimulus coding in the brain (Franke et al., 2016; Zylberberg et al., 2016). Given that we observed this relationship only for spatially familiar areas but not novel, this could indicate that novelty and familiarity may be two distinct stimuli for the hippocampus (spatial or otherwise) and are subsequently processed differently from one another.

The inverse relationship between mean firing rate and spatial information scores has been established previously in hippocampal place cells (William and McNaughton, 1992; Markus et al., 1994) and subicular cells (Simonnet and Brecht, 2019). We observed the same relationship for spatially familiar areas across days. Moreover, information scores increased across days for both common and novel segments of the tracks as overall familiarity of the environment increased, indicating they are heavily influenced by global spatial parameters (multiple stable cues in the environment such as position of distal visual cues, position of reward corner, position of common segments, and entry point of animal on track) vs. local spatial parameters (change or addition in length or geometry of spatial area, etc.).

*In vivo* data demonstrating how CA1 and CA2 interact and contribute to spatial novelty detection and encoding are sparse, with most studies focusing on either social or novel contextual changes (Wintzer et al., 2014; Alexander et al., 2016) or non-spatial aspects of memory (Mankin et al., 2015). Other studies have primarily focused on novelty detection and encoding only in CA1, in an object-place recognition task (Larkin et al., 2014), place field plasticity (Frank, 2004), or temporal coding and episodic memory (Mankin et al., 2012). Moreover, recent studies have shifted the focus of CA2’s contribution to social recognition memory and social information processing (Hitti and Siegelbaum, 2014; Meira et al., 2018; Oliva et al., 2020). However, CA2 is a well-connected, strategically located and important contributor to overall hippocampal parahippocampal memory and information processing. It not only receives direct excitatory inputs from CA3 (Chevaleyre and Siegelbaum, 2010; Kohara et al., 2014) and DG (Shinohara et al., 2012) but also EC (Bartesaghi and Gessi, 2004; Lein et al., 2007; Chevaleyre and Siegelbaum, 2010; Sun et al., 2014). It also receives extrahippocampal projections from the medial septum, diagonal band of Broca, paraventricular nucleus, and median raphe nucleus, and from regions that signal novelty such as the supramammillary nucleus (Vertes and McKenna, 2000; Cui et al., 2013; Zhang and Hernández, 2013; Hitti and Siegelbaum, 2014).

Previous studies have shown that both CA3 and CA1 place cells are less stable in novel vs. familiar environments (Leutgeb, 2004), whereby CA1 input is largely dominated by the powerful disynaptic pathway of EC–CA2–CA1 and is only later on taken over by the classic trisynaptic pathway over days (post 24 h) to stabilize CA1 place fields (Karlsson and Frank, 2008). Lesion studies on the EC–CA1 pathway have observed impaired spatial coding (Brun et al., 2008), indicating that CA2 directly influences spatial acquisition and learning-driven processes in CA1. CA2 is uniquely poised within the hippocampal circuitry and has strong unidirectional connections to CA1 (Chevaleyre and Siegelbaum, 2010) and bi-directional feed forward connections to CA3 (Kohara et al., 2014; Boehringer et al., 2017). While dorsal CA2 projections to the ventral hippocampus are required for social recognition memory (Meira et al., 2018), the same projections (excitatory) to dorsal CA1 seem to be involved in maintaining sequential firing patterns and working memory in CA1. Loss of inputs from dorsal CA2 to dorsal CA1 disrupts temporal coding in CA1 and decreases the precision and stability of CA1 time cell firing (MacDonald and Tonegawa, 2021). All these aforementioned studies establish that multiple independent circuitries coexist within the hippocampus and are activated simultaneously or alternately for different functional optimizations, with maximum influence being exerted on the most downstream subfield of the hippocampus, i.e., CA1.

Furthermore, changes in an environment such as shape (Mankin et al., 2015) and color (global changes) affect CA2 place fields the least when compared to CA1 or CA3 place fields. On the other hand, their sensitivity to smaller contextual changes and local cues (Colgin et al., 2008; Wintzer et al., 2014; Lee et al., 2015; Alexander et al., 2016) suggests that CA2 activity is an indicator of novelty signal to its downstream areas, i.e., CA1. Given that introducing novel segments in a relatively familiar spatial area on days 2 and 3 could be construed as a contextual change in the original environment with respect to spatial area, track length, and geometry, the results reported here fall in line with those reported previously. Whether in a social or a spatial aspect, novelty acts as a potent cue for hippocampal CA2 ensemble. When spatial novelty is introduced in an otherwise stable and familiar environment (on day 2 and day 3), it could act as an even more salient sensory cue due to its local context to elicit the responses from CA2 as observed in this study.

Competitive, alternate, and independent circuitries *via* CA3 (trisynaptic pathway) and CA2 (disynaptic pathway) to CA1 as well as excitatory projections from CA2 to CA3 (Kohara et al., 2014) modulate the flow of spatial information in the hippocampus depending on different behavioral states (sleep vs. awake). It has been proposed that CA2 drives sensory-based representations in the awake state, and that CA3 drives memory-based representation during sleep (Middleton and McHugh, 2020) *via* adenosine, which allows for CA3 to control sleep replay content. Thus, during the development of novel place fields, the input to CA1 is primarily dominated by CA2. This could explain the similar pattern in responses of both CA1 and CA2 with respect to their average firing rates and pairwise cross correlations for common and novel segments across days. However, given the sensitivity of CA2 to local salient cues such as novelty, spatial information scores of both ensembles differ for place cells coding for novel segments for all days. This difference in spatial information scores between CA1 and CA2 has also been reported in recent studies (Mankin et al., 2015; Alexander et al., 2016; Oliva et al., 2016; Donegan et al., 2020) strengthening this observation.

The hippocampus is needed for memorizing episodic experiences, which involve both a spatial and a temporal aspect to them. Given that dCA2 inputs to dCA1 play an important role in sequential organization of CA1 time cells (MacDonald and Tonegawa, 2021), CA2 could play a bigger role in maintaining the temporal aspect of an episodic experience more than the spatial aspect. While CA2 activity itself is affected by spatial novelty, as indicated by low spatial information content in CA2 place cells coding for a novel area, it could play a larger role in maintaining and affecting the temporal organization of CA1 place cells in sequences encountered during the experience; thereby allowing for CA1 to recognize and distinguish between familiarity and novelty in space. CA2 population responses to novel block comparisons vastly differed from CA1 responses. CA2 population correlations decreased both across days for individual novel block comparisons and within each day for successive to non-successive block comparisons. This sequential decrease in consistency of CA2 firing responses to both time and space could indicate more competent temporal processing of novel spatial experiences in CA2 than in CA1, which could then be passed on to CA1 as a temporal code.

In conclusion, CA2 inputs to CA1 play an important role in processing hippocampal-based learning and memory of spatial tasks with social, contextual (e.g., novelty), temporal, and sequential aspects. These aspects, in combination or alone, act as salient cues that modulate CA2 ensemble responses. If CA2 codes for time and CA3 for spatial context (Mankin et al., 2015), the downstream CA1 area could get a spatial-temporal map coalesced from CA3 and CA2 through different hippocampal circuitries. Moreover, temporal/sequential processing is heavily influenced and dependant on relative familiarity and novelty of episodic experiences. Given that novelty acts as a strong, salient modulator for CA2 activity compared with familiarity, it could have a direct influence on temporal organization of novel spatial experiences. Moreover, CA2 seems to be adept at distinguishing between novelty and familiarity on minor time scales (less than 10 min) and even within the same lap (on days 2 and 3), as observed in this study across all four block recordings in a single day. This study takes the first step in understanding how spatial and temporal coding of novelty takes place in the hippocampus, particularly in CA2, on multiple time scales. Finally, this study also opens up avenues where bigger, bolder questions may be asked through this paradigm to further understand spatial (novel and familiar) detection, encoding, and consolidation mechanisms in the hippocampus.

## Data Availability Statement

The datasets presented in this article are not readily available because for data and codes used in this manuscript, a detailed request may be sent to the corresponding author and as per institutional guidelines will be shared. Requests to access the datasets should be directed to GB, guncha06@gmail.com.

## Ethics Statement

The animal study was reviewed and approved by the Institutional Animal Ethics Committee (IAEC) of National Brain Research Centre at Manesar, Haryana, constituted by the Committee for the Purpose of Control and Supervision of Experiments on Animals (CPCSEA), Government of India in accordance with NIH guidelines.

## Author Contributions

GB conceived and designed the study, performed the experiments, and wrote the manuscript. GB and IN performed the experiments and the data analysis and interpretation and revised and edited the manuscript. Both authors contributed to the article and approved the submitted version.

## Conflict of Interest

The authors declare that the research was conducted in the absence of any commercial or financial relationships that could be construed as a potential conflict of interest.

## Publisher’s Note

All claims expressed in this article are solely those of the authors and do not necessarily represent those of their affiliated organizations, or those of the publisher, the editors and the reviewers. Any product that may be evaluated in this article, or claim that may be made by its manufacturer, is not guaranteed or endorsed by the publisher.

## Footnotes

1
www.mathworks.com

2
www.graphpad.com/scientific-software/prism/
