# Supplementary material for: Dynamic Hippocampal CA2 Responses to Contextual Spatial Novelty
Source: Front Syst Neurosci. 2022 Aug 8;16:923911. doi: 10.3389/fnsys.2022.923911 (PMC9393711; doi:10.3389/fnsys.2022.923911)
Supplement: Supplementary file 1 [file Data_Sheet_1.pdf]

Supplementary figure legends

Supplementary Figure 1

A. Pie charts showing percentages of CA1 and CA2 place cells that fired in all 4 block sessions within a day's recording, those that fired in 3 out of 4 blocks, in 2 blocks or only in 1 block session. Majority of place cells from both ensembles fired in at least 3 out of the 4 recording blocks in a day.

B. Place cell distributions for CA1 and CA2 for common and novel segments of the tracks on day 2 and 3, depicted for each animal individually. Across all animals, both CA1 and CA2 had similar place cell distribution for cells that fired for common and novel segments of the track (except rat 3 for CA2). On day2, a higher number of place cells fired for novel segments (in red) than common (blue) for both ensembles. However, the reverse was seen on day 3 with higher number of place cells firing for common segment of the track than the novel segment. * indicates $p<.05$, Chi square test: CA1 + CA2 cells: common vs novel: day2: $p=.0142$, day3: $p=.0253$; CA1 cells: day2: $p=.0253$, day3: $p=.0367$).

Supplementary Figure 2

A. Average speeds were compared for all animals within a particular day, over all block recordings and no significant variability was observed for any particular animal or a particular recording session for any day of the experiment (Kruskal Wallis test for block 1 vs block 2 vs block 3 vs block 4: RUN1: $p=.797$, RUN2: $p=.795$ and RUN3: $p=.543$)

B. Comparison of average running speed of all animals between block1 (1st 20 laps) and block 4 (last 20 laps for the day) for all days. All animals (except rat 4 on day1) showed an increase in average speed from block1 to 4, indicating that the animals learnt the experimental paradigm over time on all days (Jonckheere Terpstrsa test: block 1 vs 4 across all days: p=.065)

C. Average running speeds were averaged across all 4 block sessions within a day and compared across days for all animals. Speeds increased across days for all animals, indicating learning occurred both within a day as well as across days as well, with increasing overall familiarity of the environment, despite the length and geometry of the tracks changing each day. (Kruskal Wallis test: day1 vs 2 vs 3: p=.0488)

Supplementary Figure 3

Histology: Representative examples of 40 μm thick nissl stained coronal sections from the hippocampus showing clear tetrode traces in both CA1 and CA2 from all recorded animals are displayed. Alongside each histological section, a corresponding schematic section from Rat Paxinos Atlas at the same A-P coordinates is shown. This is done particularly to establish CA2 borders conclusively, as CA2 is an extremely small region between CA1 and CA3 and its antero-posterior position varies along the axis throughout the rat hippocampus. CA2 borders are also marked in black in each histological slice as well, based on the borders defined in each corresponding Atlas section below it. Tetrode traces in CA1 and CA2 are marked with black arrows in each histological slice.

Supplementary Figure 4

A. Violin plots for of CA1 and CA2 place cells comparing average firing rates for common and novel segments over all 4 block sessions. While within a day, no trend was observed for firing rates across the 4 blocks for either common or novel segments; it was observed that within each block session, average firing rates decreased across days for common segments but not for novel segments. (* indicates p<.05, ** indicates p<.005 and *** indicates p<.0005; Jonckheere Terpstrsa test: common segments: CA1 cells: p=.001, CA2 cells: p=.002; novel segments: CA1 cells: p=.120, CA2 cells: p=.152)

B. Average firing rates were computed for each arm of common and novel segments of all tracks for CA1 and CA2 populations. The novel segments are depicted in red while common segments are depicted in blue. While on day2, average firing rates were marginally higher for novel arms in comparison to familiar arms, they were significantly higher on day 3. This was observed for both ensembles separately. (Mann Whitney test: common vs novel: day2: CA1 cells: p=.292, CA2 cells: p=.694; day3: CA1 cells: p=.001; CA2 cells: p=.0198).

Supplementary Figure 5

A. Violin plots for of CA1-CA1 and CA1-CA2 cell pairs comparing pairwise cross correlations for common and novel segments over all 4 block sessions. No trend was observed over 4 blocks within a day, for any of the days. However, within each block session, pairwise cross correlations decreased for common segments but not for novel segments, for both ensembles (except for novel CA1-CA2 pairs probably due to less number of cells for novel segments on day 3). (** indicates

p<.005 and *** indicates p<.0005; Jonckheere Terpstrsa test: common segments: CA1-CA1 pairs: p=.0017, CA1-CA2 pairs: p=.001; novel segments: CA1-CA1 pairs: p=.441, CA1-CA2 pairs: p=.06).

B. Pairwise cross correlations were computed for each arm of common and novel segments of all tracks for CA1 and CA2 populations. The novel segments are depicted in red while common segments are depicted in blue. While on day2, average firing rates were marginally higher for novel arms in comparison to familiar arms, they were significantly higher on day 3. This was observed for both ensembles separately. However, due to less number of CA2 place cells for novel segment on day3, the test could only be performed for CA1. (Mann Whitney test: common vs novel: day2: CA1 cells: p=.364, CA2 cells: p=.149; day3: CA1 cells: p=.00049).

Supplementary Figure 6

A. Violin plots of spatial information scores for CA1 and CA2 place cells compared across common and novel segments within each block recording. Contrary to the trend observed for average firing rates and pairwise cross correlations, spatial information scores increased for both common and novel segments for CA1 and CA2 across days. (** indicates p<.005 and *** indicates p<.0005; Jonckheere Terpstrsa test: common segments: CA1 cells: p=.0003, CA2 cells: p=.00038; novel segments: CA1 cells: p=.0003, CA2 cells: p=.0017).

B. Spatial information scores were computed in each arm of common and novel segments for CA1 and CA2 populations. The novel arms are depicted in red while

familiar arms are depicted in blue. Unlike average firing rates and pairwise cross correlation comparisons, within day comparison for day 2 and 3 revealed no distinction between information scores of place cells on novel arms vs familiar arms for either CA1 or CA2 (Mann Whitney test: common vs novel: day2: CA1 cells: p=.692; CA2 cells: p=.628; day3: CA1 cells: p=.687, CA2 cells: p=.378)
