# Supplementary material for: Dynamic Hippocampal CA2 Responses to Contextual Spatial Novelty
Source: Front Syst Neurosci. 2022 Aug 8;16:923911. doi: 10.3389/fnsys.2022.923911 (PMC9393711; doi:10.3389/fnsys.2022.923911)
Supplement: Supplementary file 2 [file Data_Sheet_2.PDF]

Supplementary materials:

Supplementary figure 1:

Hippocampal place cell counts:

A: All place cells from CA1 and CA2 that fired on either common or novel segments of the tracks were tracked throughout the day (over 4 block sessions) and it was determined that majority of place cells fired in at least 3 out of all 4 block recordings.

B: Place cell distribution over common and novel segments of the track on days 2 and 3 were assessed individually for each animal (based on place cell distribution observed in figure 1D). It was observed that on day 2, a higher number of place cells fired for novel segments, from both CA1 and CA2. Conversely, the opposite was observed on day 3, where a higher number of CA1 and CA2 place cells fired for common segment of the track (except rat 3 for CA2). (Chi square test: common vs novel: all place cells together: day2: p=.0142, day3: p=.0253; CA1 cells: day2: p=.0253, day3: p=.0367). Note: the test was done for all place cells together (CA1 + CA2) and for CA1 cells separately. However, CA2 place cell quantity was too small to observe differences through this test, even though 3 out of 4 rats displayed the same trend as CA1 on both days 2 and 3.

Supplementary figure 2:

Animal behaviour and learning:

Since the experimental paradigm is completely new, we checked for animal behaviour and learning both across days and within each day. For the same, we assessed average running speed of all animals, which is indicative of how well versed the animal is with the paradigm

and its surrounding environment, while also acknowledging and understanding the paradigm at hand.

A: The average running speed of all animals were computed in individual block recordings throughout the day, for all days. They were then compared for all animals within a particular day, over each block recording separately, and no significant variability was observed for any particular animal or a particular recording session for any day of the experiment (Kruskal Wallis test for block 1 vs block 2 vs block 3 vs block 4: day1: $p=.797$, day2: $p=.795$ and day3: $p=.543$)

B: Next, we compared the average speeds for all animals over block 1 (most novel block of the day) and block 4 (most familiar block of the day) within a day, over all 3 days and observed that 3 out of 4 animals had higher average speeds in block 4 from day 1 itself, indicating that the animals were learning from the 1st block session of the experimental paradigm itself. The 4th animal (rat 4), eventually on day 3 had higher average speed from block 1 to 4 on day3, indicating slower learning compared to other animals, but learning nonetheless (Jonckheere Terpstrsa test: block 1 v/s block 4: $p=.065$, excluding rat 4 on days 1 and 2)

C: Lastly, we averaged the running speed across all 4 blocks within a day and compared the average speeds across days for all animals. It was found that the average speeds significantly increased over days for all animals, indicating that they grew accustomed to the environment and its overall increasing familiarity, despite novel segments being added to the tracks on multiple days, while also learning the paradigm well (Kruskal Wallis test, right tailed with post hoc Bonferonni correction: average speeds for day1 vs day2 vs day3 for all animals $p=.0488$).

Supplementary figure 3:

Histology:

Representative examples of 40 µm thick Nissl-stained coronal sections from the hippocampus showing clear tetrode traces in both CA1 and CA2 from all recorded animals are displayed. Alongside each histological section, a corresponding schematic section from Rat Paxinos Atlas (Paxinos and Watson, 2007) the same A-P coordinates is shown. This was done particularly to establish CA2 borders conclusively, as CA2 is an extremely small region between CA1 and CA3 and its antero-posterior position varies along the axis throughout the rat hippocampus. CA2 borders also marked in black in each histological slice as well, based on the borders defined in each corresponding Rat Atlas section below it. Tetrode traces in CA1 and CA2 are marked with black arrows in each histological slice. Detailed methodology of Nissl staining and the histology procedure followed is given in the materials and methods section of the manuscript.

Supplementary figure 4:

Average firing rates:

A: A decrease in average firing rates was observed across days for common segments (figure 2A). To check if the same trend would be observed within each day as well, firing rates were calculated for all 4 blocks within a day, for all days. No trend was observed across 4 blocks within a day, for any of the recording days, for either common or novel segments, for either CA1 or CA2 populations. However, within each block, firing rates decreased across days for common segments, but not for novel segments. Thus, the same trend observed in figure 2A, was observed over all 4 individual blocks as well. The same was observed in both CA1 and CA2 populations separately (Jonckheere Terpstrsa test: day1 vs day2 vs day 3: common segments: CA1 cells: p=.001, CA2 cells: p=.0002; novel segments: CA1 cells: p=.120, CA2 cells: p=.152)

This further cements the previous observation that as a spatial area gets more familiar, the average firing rates decrease over time. Black solid bars indicate median of the group.

B: Average firing rates were also computed for each arm individually of the common and novel segments for all 3 days, for CA1 and CA2 separately. Within days' comparison between common and novel segments on day 2 and day 3 revealed interesting differences. On both days, place cells for novel segments had higher firing rates than the common segments, revealing the same trend. However, those differences were only marginally higher on day 2 for both populations but were significantly higher on day 3. This remained true for both hippocampal populations. (Mann Whitney U test: common vs novel segments: day2: CA1 cells: p=.292, CA2 cells: p=.694; day3: CA1 cells: p=.0001; CA2 cells: p=.0219).

Supplementary figure 5:

Pairwise cross correlations:

A: As was observed with average firing rates, pairwise cross correlations also decreased across days for common segments (Figure 2B). However, cross correlations across 4 blocks within a day did not reveal any trend for common or novel segments on any of the days for either CA1 or CA2. Instead, similar to the trend observed for firing rates, cross correlations for both CA1-CA1 and CA1-CA2 decreased across days within each block for common segments, but no such trend was observed for novel segments, (with the exception of novel CA1-CA2 cell pairs, that also showed a decreasing trend over days, probably due to less number of CA1-CA2 pairs for novel segments on day 3). (Jonckheere Terpstrsa test: day1 vs day2 vs day 3: common segments: CA1-CA1 pairs: p=.0017, CA1-CA2 pairs: p=.001; novel segments: CA1-CA1 pairs: p=.441, CA1-CA2 pairs: p=.06). Black solid bars indicate median of the group.

B: Pairwise cross correlations for CA1 and CA2 cell pairs were computed in each arm

individually for common and novel segments across all days**.** Within days' comparison for the day2 and day3 revealed the same trend as was observed for firing rates. On day 2, both cell pairs had marginally higher cross correlations for novel segments. However, on the 3rd day, they were significantly higher in comparison to common segments (Mann Whitney U test: common vs novel segments: CA1-CA1 pairs: day2: p=.364, day3: p.00049; CA1-CA2 pairs: p=.149; CA1-CA2 pair comparison could not be done for day 3 due to no pairs in arm J and very less pairs in arm I).

Supplementary figure 6:

Spatial information score:

A: Contrary to the trend observed for average firing rates (Figure2A) and pairwise cross correlations (Figure2B), spatial information scores increased across days for both common and novel segments of the tracks (Figure 3A). This same trend was observed over each block as well (Jonckheere Terpstrsa test: day1 vs day2 vs day3: common segments: CA1 cells: p=.0003, CA2 cells: p=.00038 and novel segments: CA1 cells: p=.0003, CA2 cells: p=.0017). This was the opposite trend of that observed for average firing rates and pairwise cross correlations, especially with respect to comparing the familiar parts of the maze. However, no trend was observed for any day, across 4 blocks within a day for either common or novel segments. Black solid bars indicate median of the group.

B: Spatial information scores were also computed for each arm for all segments for all days. Within day comparison between common and novel segments for day 2 and 3 revealed no distinction between information scores for either CA1 or CA2 (Mann Whitney U test: common vs novel: day2: CA1 cells: p=.692; CA2 cells: p=.628; day3: CA1 cells: p=.687, CA2 cells: p=.378). Therefore, within the same closed loop track, while average firing rates and pairwise cross correlations were lower for familiar area in comparison to novel area, information scores

remained similar and comparable for both areas.

Supplementary figure 7:

List of statistical tests used in the study:

A detailed list of various statistical tests used throughout the study are listed in the table.

References:

Paxinos, G., and Watson, C. (2007). *The Rat Brain in Stereotaxic Coordinates*. 6th edition. Elsevier/academic.
