# Supplementary figures and images for: Dynamic Hippocampal CA2 Responses to Contextual Spatial Novelty
Source: Front Syst Neurosci. 2022 Aug 8;16:923911. doi: 10.3389/fnsys.2022.923911 (PMC9393711; doi:10.3389/fnsys.2022.923911)

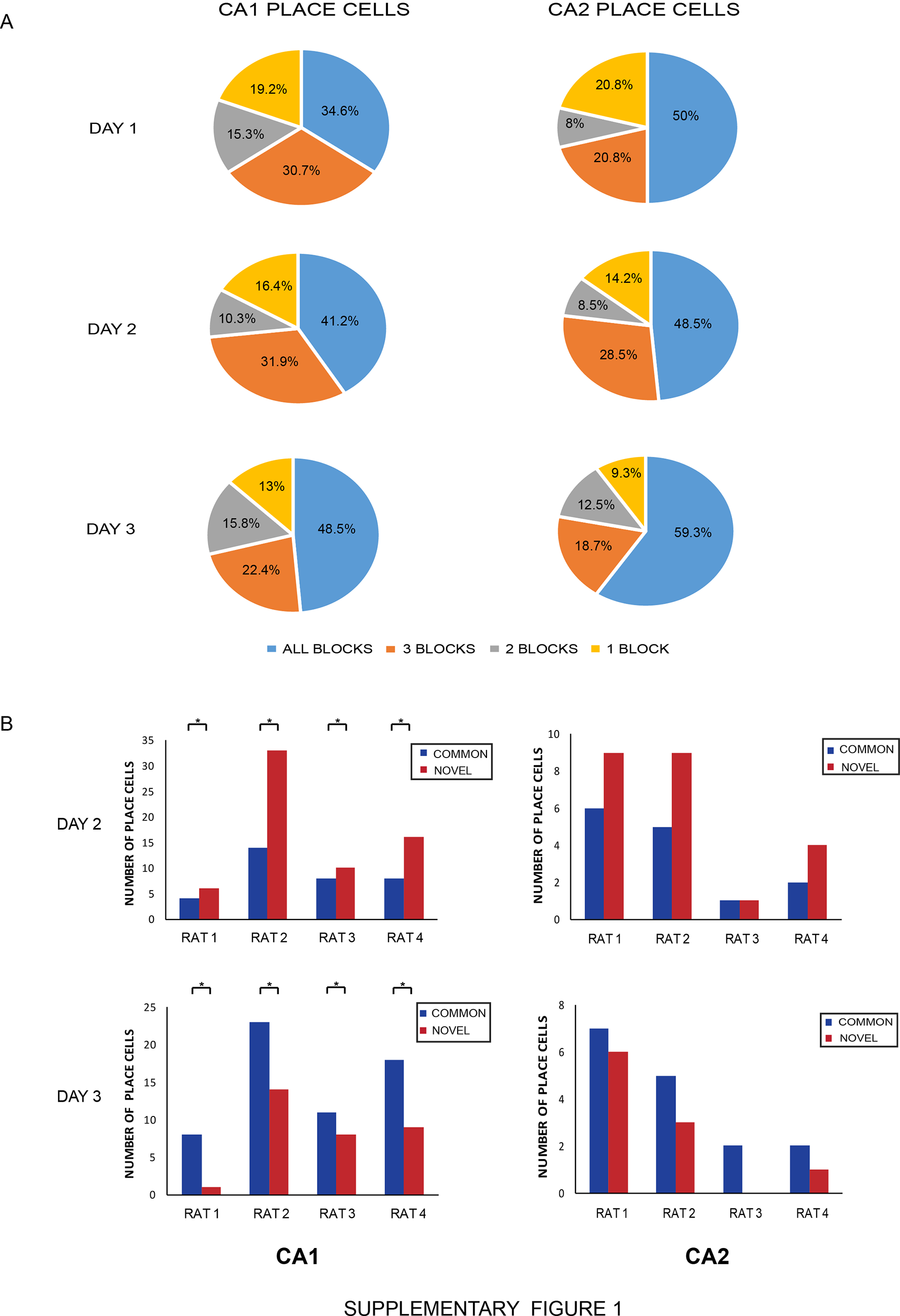

Supplement: Supplementary file 3 [file Image_1.tif]

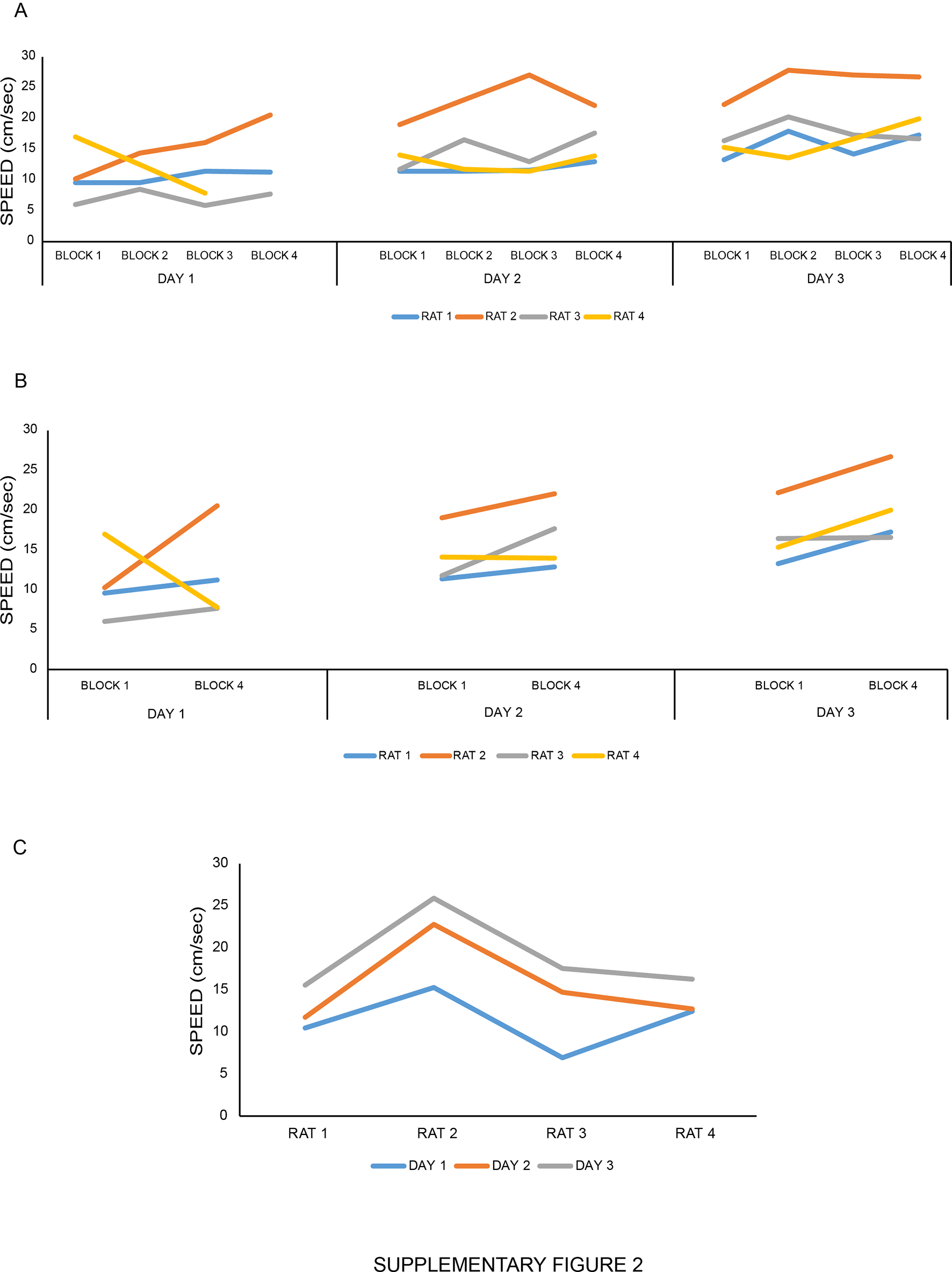

Supplement: Supplementary file 4 [file Image_2.tif]

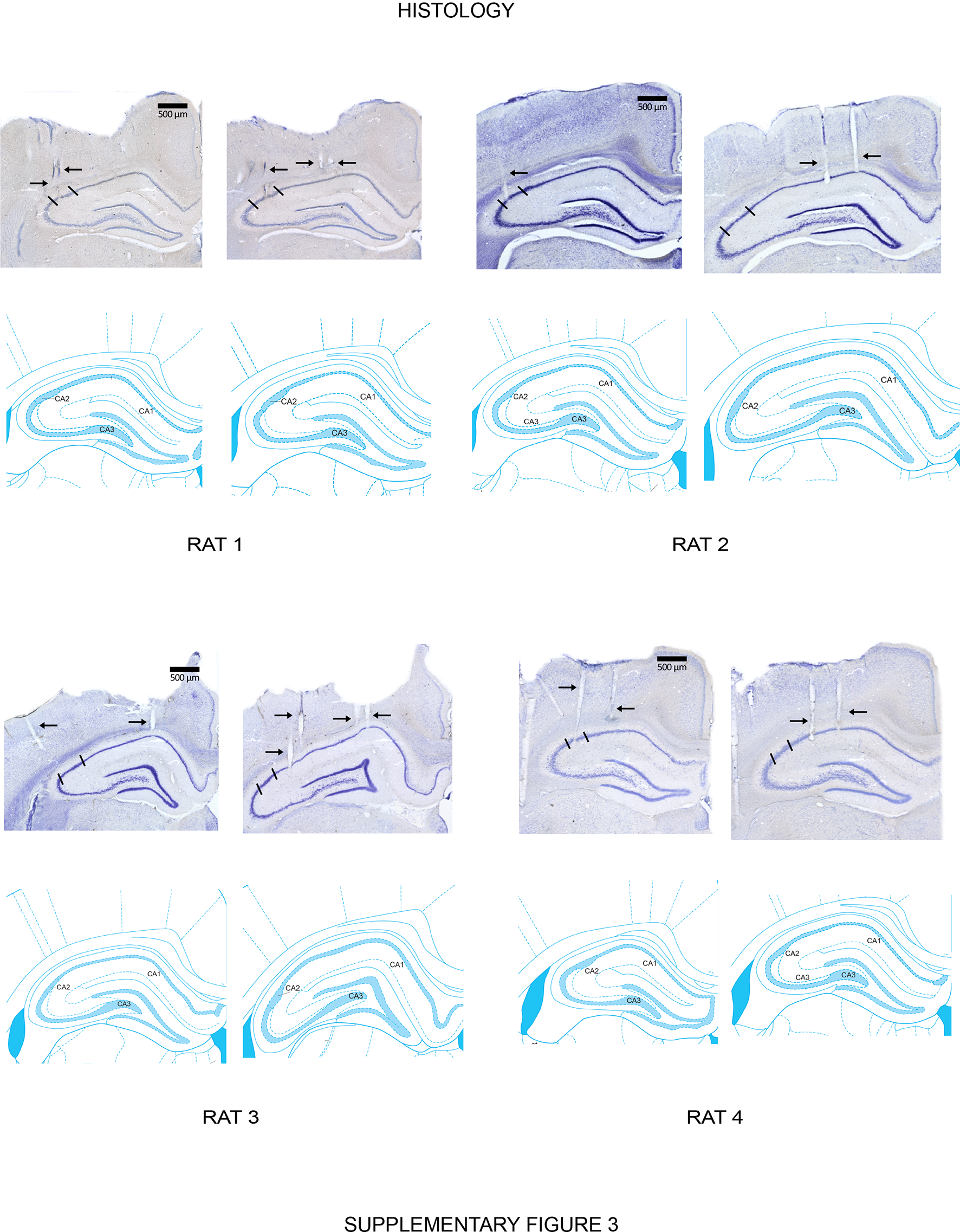

Supplement: Supplementary file 5 [file Image_3.tif]

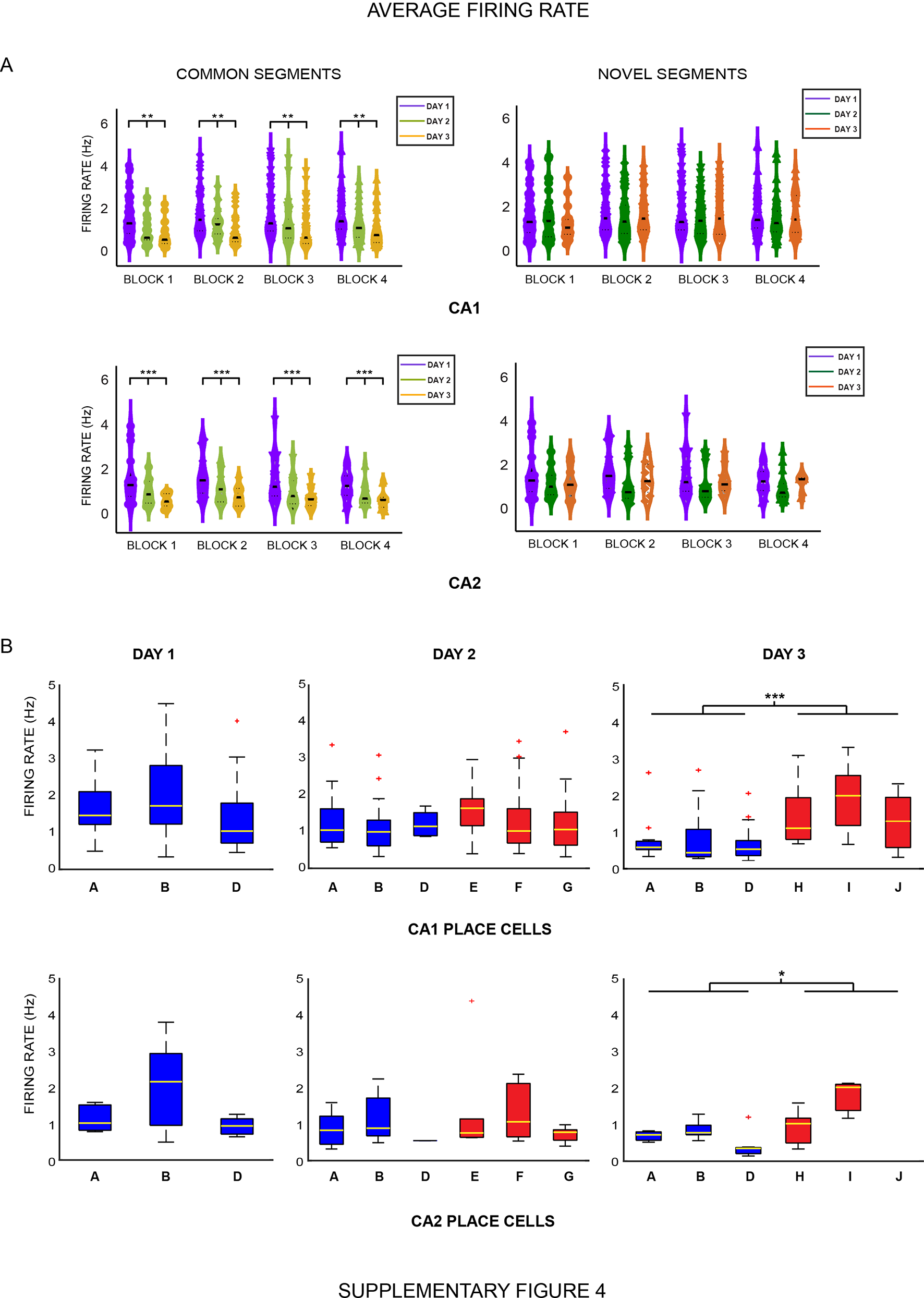

Supplement: Supplementary file 6 [file Image_4.tif]

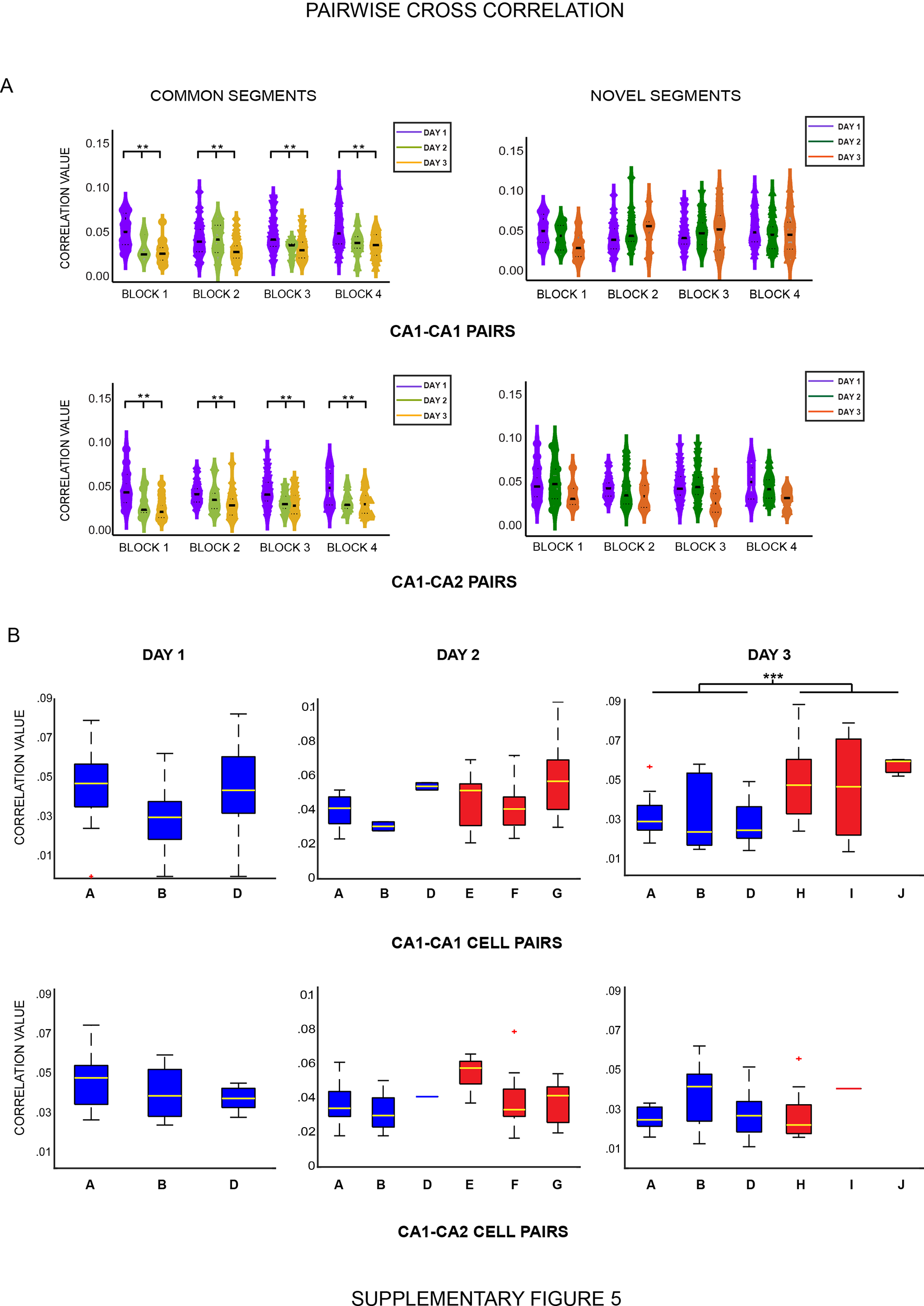

Supplement: Supplementary file 7 [file Image_5.tif]

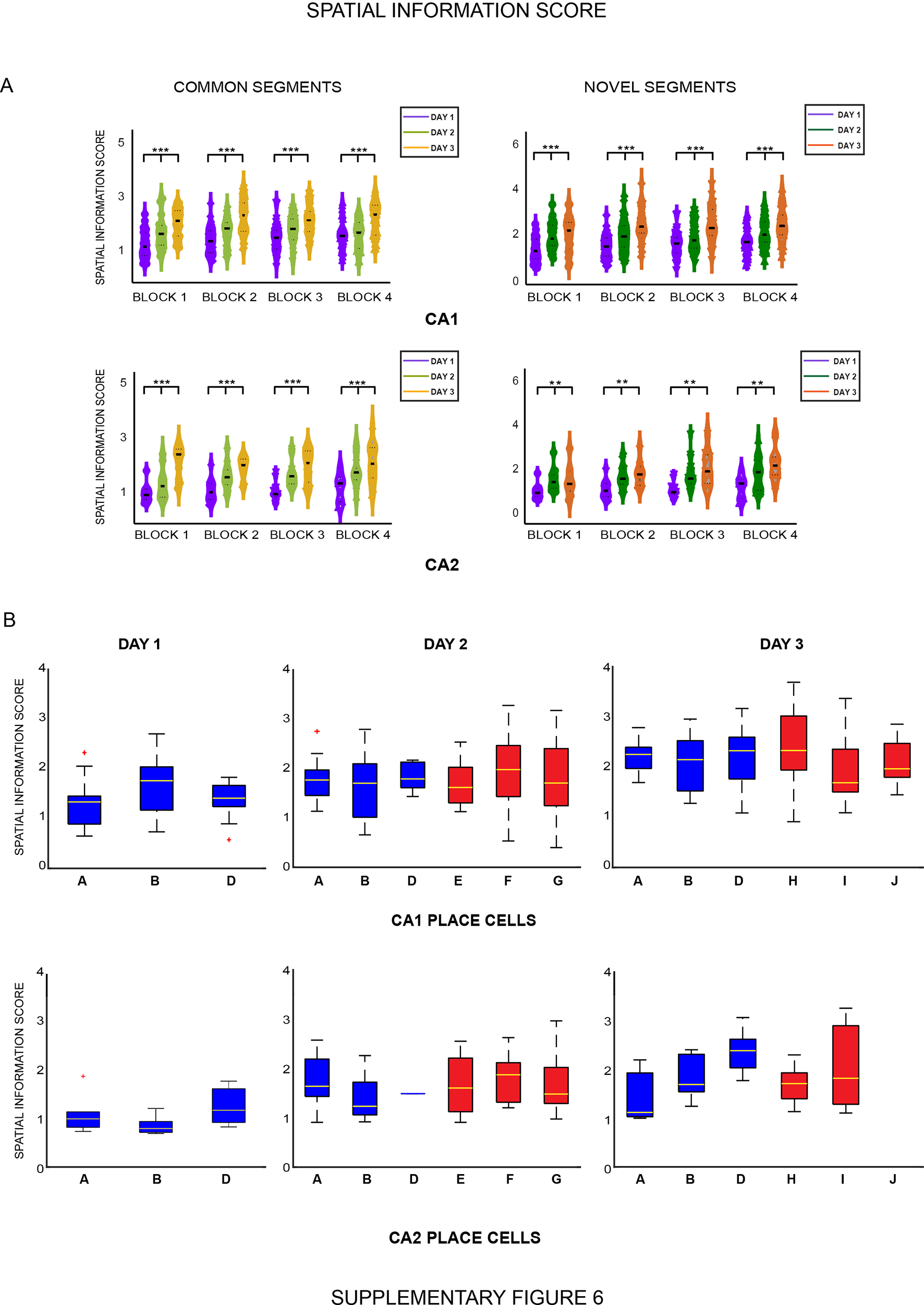

Supplement: Supplementary file 8 [file Image_6.tif]

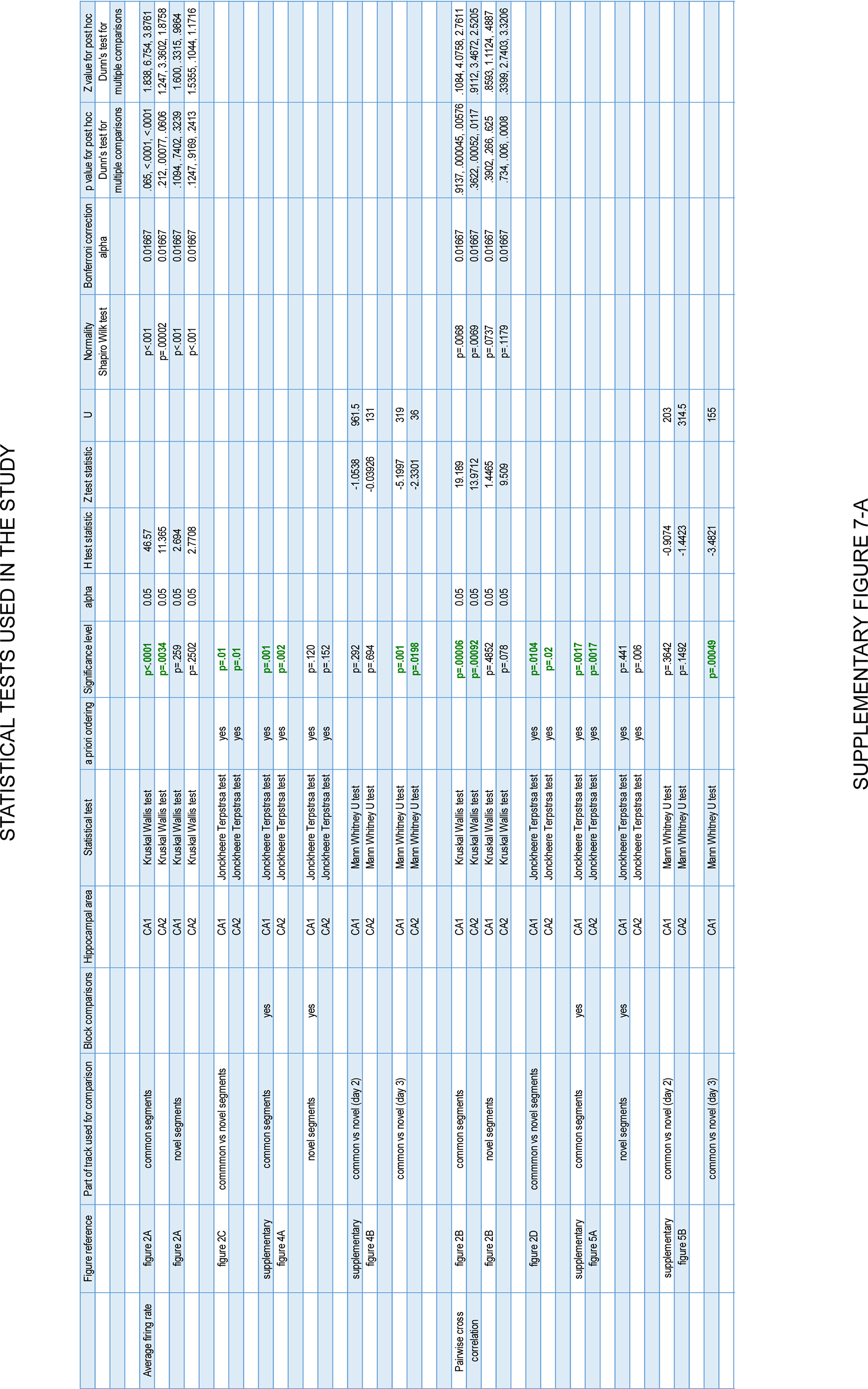

Supplement: Supplementary file 9 [file Image_7.tif]

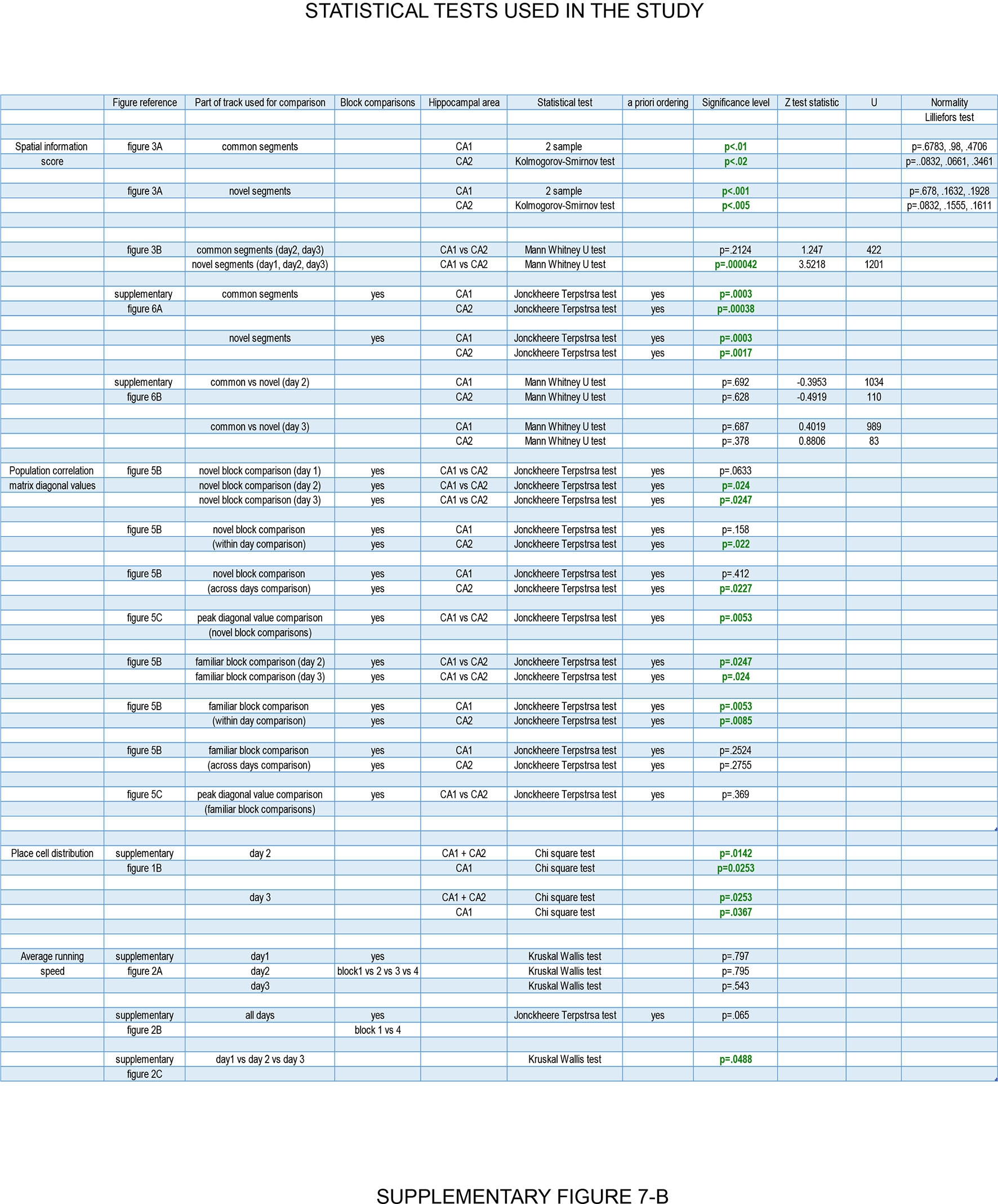

Supplement: Supplementary file 10 [file Image_8.tif]
